# The effect of sleep restriction on empathy for pain: An fMRI study in younger and older adults

**DOI:** 10.1038/s41598-017-12098-9

**Published:** 2017-09-25

**Authors:** Sandra Tamm, Gustav Nilsonne, Johanna Schwarz, Claus Lamm, Göran Kecklund, Predrag Petrovic, Håkan Fischer, Torbjörn Åkerstedt, Mats Lekander

**Affiliations:** 1 Department of Clinical Neuroscience, Karolinska Institutet, Stockholm, Sweden; 2 Stress Research Institute, Stockholm University, Stockholm, Sweden; 3 Department of Psychology, Stockholm University, Stockholm, Sweden; 4 Social, Cognitive and Affective Neuroscience Unit, Department of Basic Psychological Research and Research Methods, Faculty of Psychology, University of Vienna, Vienna, Austria; 5 Behavioural Science Institute, Radboud Universiteit, Nijmegen, Netherlands

## Abstract

Age and sleep both affect emotional functioning. Since sleep patterns change over the lifespan, we investigated the effects of short sleep and age on empathic responses. In a randomized cross-over experimental design, healthy young and older volunteers (*n* = 47 aged 20–30 years and *n* = 39 aged 65–75 years) underwent functional magnetic resonance imaging (fMRI) after normal sleep or night sleep restricted to 3 hours. During fMRI, participants viewed pictures of needles pricking a hand (pain) or Q-tips touching a hand (control), a well-established paradigm to investigate empathy for pain. There was no main effect of sleep restriction on empathy. However, age and sleep interacted so that sleep restriction caused increased unpleasantness in older but not in young participants. Irrespective of sleep condition, older participants showed increased activity in angular gyrus, superior temporal sulcus and temporo-parietal junction compared to young. Speculatively, this could indicate that the older individuals adopted a more cognitive approach in response to others’ pain. Our findings suggest that caution in generalizability across age groups is needed in further studies of sleep on social cognition and emotion.

## Introduction

Empathy is a social skill needed for interaction with other people. Empathy can be defined as spontaneously sharing another person’s feeling while being aware of the source of the emotion^1^. This function is vital in people-oriented occupations. Many of these occupations, such as healthcare or police, involve sometimes irregular work hours and sleep loss, a potential cause of disturbed emotions^2,3^. Whether social emotions, such as empathy, are affected by sleep loss, is however not known. The brain networks underlying empathy are well-studied mainly in younger adults, and the same holds true for most research on sleep and emotion. In spite of changes in both sleep^4^ and emotional functioning in elderly humans^5,6^, the role of adult aging in sleep-induced emotional modulation is uncharted.

Sleep restriction in young adults has been shown to be associated with increased amygdala activation in response to negative stimuli^3,7,8^, and decreased functional connectivity between the prefrontal cortex and amygdala^3^, suggesting decreased capacity for emotional control. Because sleep restriction affects emotional processes engaged during social emotions^2,8^, an effect of disturbed sleep on empathy can be expected. A common model for empathy involves shared emotions^9,10^ and several studies have demonstrated that seeing another person in pain leads to a subjective emotional response and activates the anterior insula (AI) and anterior midcingulate cortex (aMCC) bilaterally^9^. The network involved in empathy partially overlaps with processing of self-experienced pain^10,11^, but possibly with a different underlying pattern^12^. In the early study by Singer *et al*., activity in anterior insula was found to be correlated to self-rated trait empathy^10^, but few other studies have replicated this correlation^9,13^. Activation of self-related systems is believed to allow emotional understanding of others, and to contribute to successful social interaction^14^. In spite of high relevance in both occupational and social settings, only one experimental study has studied sleep and empathy, reporting decreased self-reported empathy after sleep restriction^15^. No study has to our knowledge investigated effects of sleep restriction on neural substrates of empathy, nor the impact of age in this context.

Older adults sleep less compared to younger, and sleep disturbances are more common in the elderly^16^. Conversely, older individuals are less affected by day-time sleepiness after sleep restriction^17^. In sleep restriction studies, older participants have shown less effects of restricted sleep on cognitive outcomes^18^. This could be true for emotional outcomes as well, but no study has so far investigated the interaction between aging, sleep, and social emotions.

During healthy aging, changes in emotional functioning are observed, such as decreased capacity to detect emotions in others, and positive bias in attention and memory^5,6^. Behavioural studies suggest an increase in empathic capacity in older individuals^19,20^. Results are however inconclusive^21,22^, and studies that use physiological outcomes are lacking. One functional magnetic resonance imaging (fMRI) study investigated emotional empathy for pain in 3 age groups (age: 20–35, 40–55, 65–80), claiming evidence for empathy-related decreased activity in AI and aMCC with older age^23^.

As manifested in behavioural as well as neural measures, both age and sleep thus affect many emotional processes^3,24^. However, associations between sleep and empathy, as well as the interaction between sleep and age in relation to empathy, are almost entirely lacking. The present study thus aimed to investigate the interplay between age and sleep restriction on self-reported and neural responses to pain in others. We hypothesized that sleep restriction causes decreased ratings of vicarious unpleasantness and decreased responses in AI and aMCC to others’ pain. In the preregistration (see below), this was formulated as follows: 1. Partial sleep deprivation (PSD) will cause decreased ratings of unpleasantness, 2. Pain stimuli will cause greater BOLD responses in the anterior insula (AI) and anterior/middle cingulate cortex (ACC/MCC) than control stimuli and PSD will interact with this increase to cause lesser increases to pain stimuli. Given the sparsity of previous research at study preregistration, no specific hypotheses regarding expected differences between younger and older participants in empathic responses, and age*sleep restriction effects on empathy, were registered. A complete list of preregistered hypotheses addressed in this manuscript is presented in the supplement.

## Methods and Materials

### Design

In a randomized cross-over design, healthy volunteers underwent MRI scanning on two occasions approximately one month apart after normal sleep and sleep restriction in a counter-balanced fashion (see below). In the sleep restriction condition, participants were instructed to sleep 3 hours in the end of their normal sleep period. MRI scanning took place in the following evening, starting between 5 PM and 8 PM. The study was carried out as part of a larger project, see ref.^25^, with data collected between 121203 and 140429. At each occasion, the experiment lasted for about three hours and included three different emotional paradigms, as well as two resting state sessions, see ref.^25^ for details. The experiment reported here was performed after an experiment on emotional mimicry and before an emotional regulation task for all participants. The study was approved by the Regional Ethics Review board of Stockholm (2012/1870-32) and preregistered at clinicaltrials.gov (NCT02000076) with a separate hypotheses list published at Open Science Framework (https://osf.io/bxfsb/), where the present experiment is referred to as “HANDS”. Experiments were performed in accordance with the Declaration of Helsinki and applicable local regulations. Preliminary results from this study have been previously reported^26–28^.

### Participants

**Figure 1:**
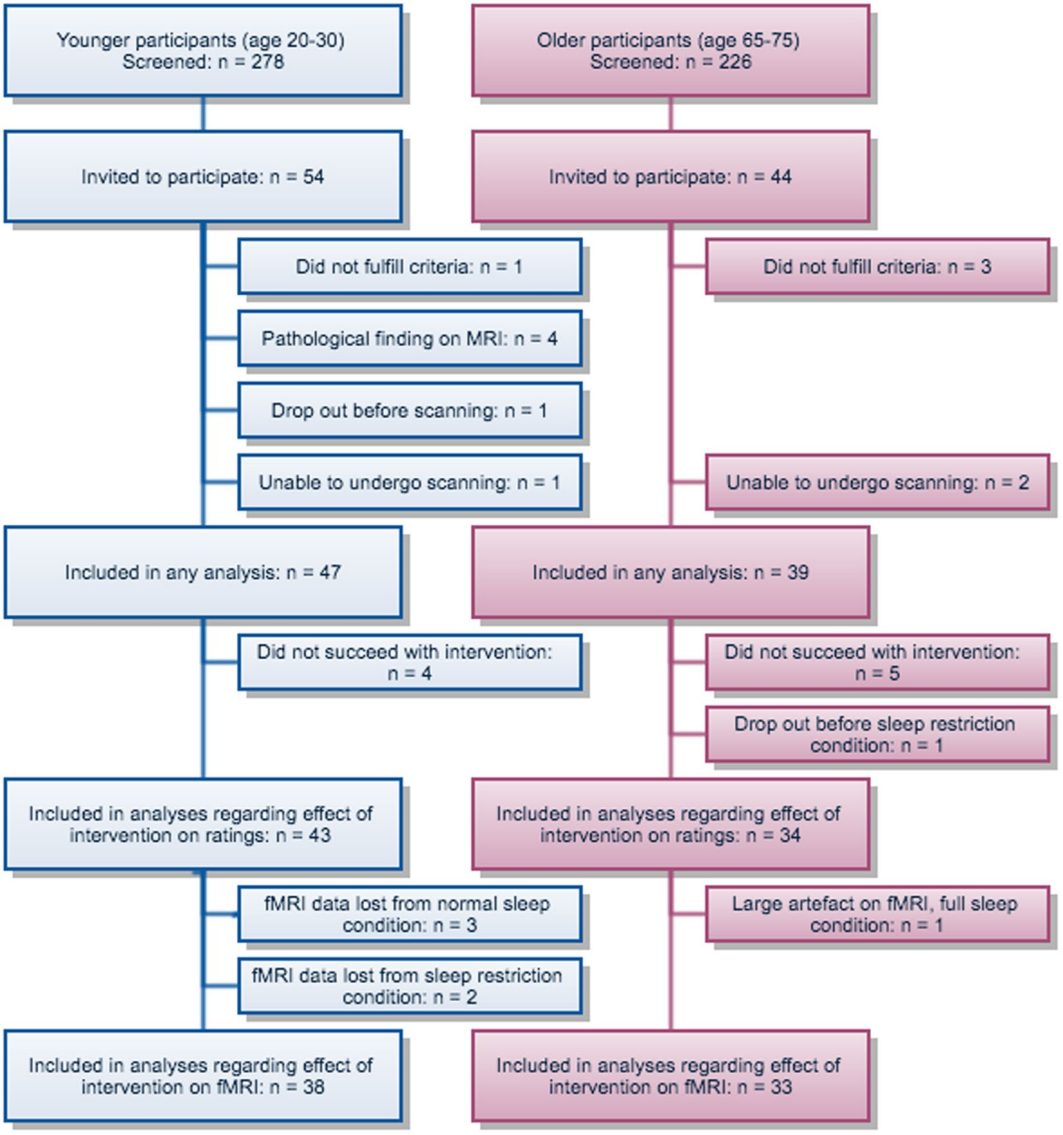
Inclusion flowchart showing participants screened and enrolled in the experiment. 278 young and 226 old participants were screened and 54 young and 44 old were enrolled in the experiment. One young participant and 3 old participants were excluded during the experiment after we discovered that they did not fulfil criteria. Four young participants were excluded from all analyses due to pathological findings on MRI. One young participant cancelled her participation after sleep intervention due to a headache. One young participant panicked in the scanner and 2 old participants were unable to undergo the experiment due to claustrophobia. We aimed for 30 participants with complete data in each group, and there was more data loss/drop out in the younger group.

Participants were recruited through the website www.studentkaninen.se, by posters at university campuses, and by a newspaper advertisement. We started recruiting young participants in December 2012. We used the following inclusion criteria: age 20–30 or 65–75 years, right-handedness, habitual bedtime between 22:00 and 01:00 on weekdays, adequate visual acuity (or mild hyperopia/myopia <5 dioptres), fluent in Swedish and living in the Stockholm area. We also used the following exclusion criteria: current or past psychiatric or neurologic morbidity, regular use of nicotine, studying or working in medicine, psychology, health care or behavioural sciences, consumption of >4 cups of coffee (or a corresponding amount of caffeine) per day, magnetic implants, colour blindness, diabetes, hypertension, use of any psychoactive drugs, self-reported claustrophobia or past heart surgery. Participants were also screened using the Insomnia Severity Index (ISI) and the Hospital Anxiety and Depression Scale (HADS) and excluded if they had ISI ≥ 15 or HADS-Depression ≥8. When we started recruiting older participants (in October 2013), we added the exclusion criterion self-reported snoring or sleep apnea symptoms more than 3 times a week. Since obstructive sleep apnea syndrome (OSAS) disturbs sleep and is more common in older people, not screening for this would have possibly introduced a confounding factor. Before that, one young participant, who reported snoring regularly 3–4 times a week, was included and was not subsequently excluded from analyses. Criteria were verified when participants arrived for the first MRI scanning session. Forty-seven young and 39 older participants were included in at least one analysis (Fig. 1). All participants gave written informed consent, and were compensated with a minimum of 2500 SEK (approximately 300 USD). Thirty-eight young and 33 older participants were included in all analyses, see below.

### Rating scales and demographic measures

Participants were characterized using rating scales and demographic measures^25^. Interpersonal Reactivity Index (IRI)^29,30^ and Psychopathic Personality Inventory-Revised (PPI-R)^31^ were used at baseline to characterize trait empathy and psychopathy. The Swedish translation of the IRI has been validated^32^, and we investigated the subscale empathic concern (IRI-EC) as a predictor for empathic responding. The rationale behind this was that in the Swedish validation, confirmatory factor analysis showed that the four subscales only distribute over two factors, one of which consists of the empathic concern subscale^32^. For PPI-R we used the subscale coldheartedness. Participants rated their sleepiness several times during the experiment using the Karolinska Sleepiness Scale^33^ and for this experiment we used the sleepiness rating immediately following the fMRI paradigm.

### Randomization and sleep intervention

Participants were randomized in blocks of 4 to have sleep restriction on the first or second night. In the final sample, 26 of the younger and 20 of the older participants started with the full sleep condition and 21 of the younger and 19 of the older started with the sleep restriction condition. During the nights before both fMRI sessions, polysomnography was recorded in the homes of the participants^25^ (results reported in Åkerstedt, submitted). Participants were instructed to either sleep as usual or for 3 hours before their normal time of rising, respectively. Successful intervention was defined by total sleep time >4 hours in the full sleep condition, <4 hours in the sleep restriction condition, and a difference between the two conditions >2 hours. Four young participants and 5 old participants did not fulfil these criteria and were therefore not included in analyses of the effect of sleep restriction. For 4 young eligible participants and 4 old eligible participants, these criteria could not be based on polysomnography, because of incomplete data. For these, subjective reports of sleep length (sleep diaries) were used. Researchers performing fMRI were blinded to participants’ sleep condition.

### fMRI paradigm and behavioural data acquisition

**Figure 2:**
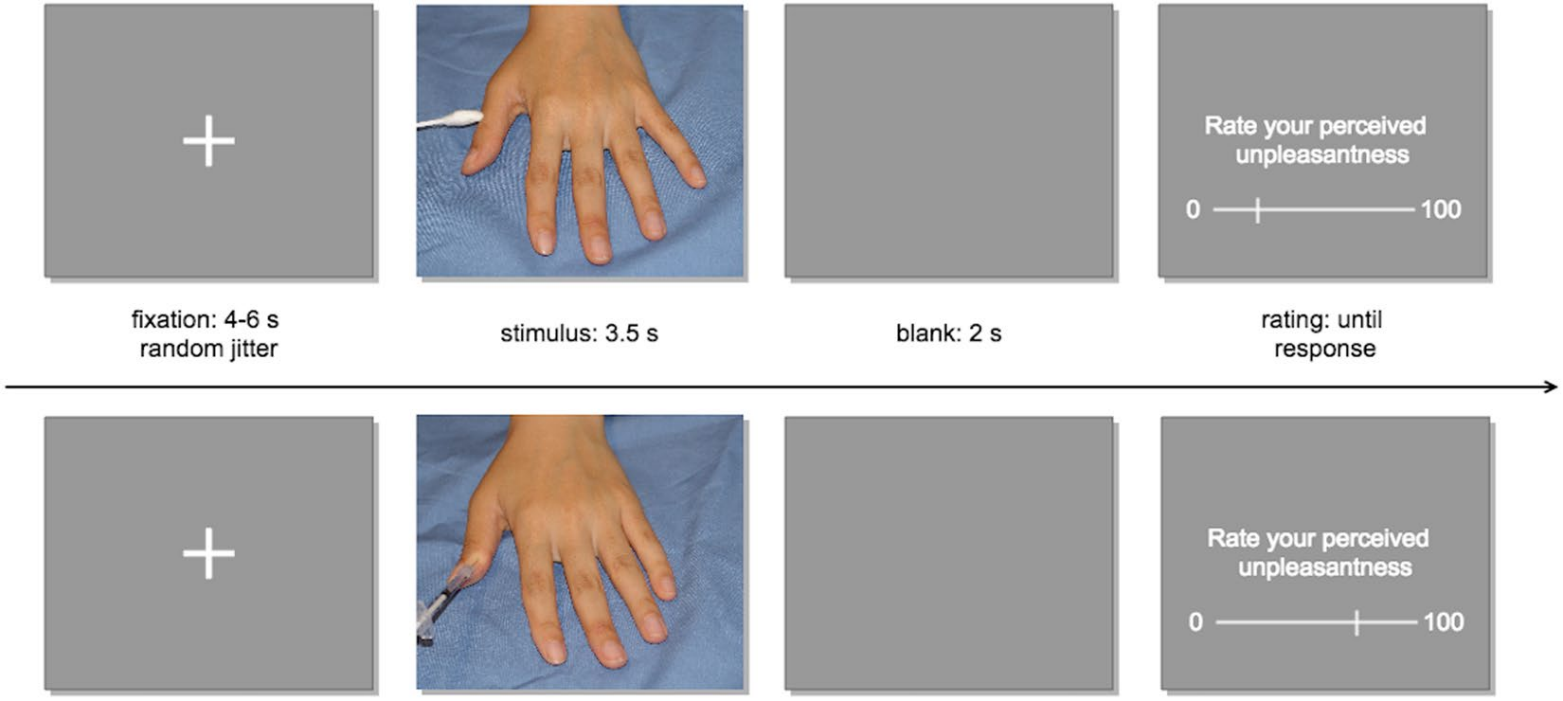
fMRI task. Stimuli showing either pain (needles) or no pain (Q-tips) were shown for 3.5 seconds and perceived unpleasantness was rated after each stimulus.

Participants were shown 20 colour pictures of hands being pricked by needles and 20 pictures of hands being touched with a Q-tip (Fig. 2). This is a well-established stimulus set used in previous studies of empathy for pain^34,35^ and we will refer to the stimuli as “pain” and “no pain” respectively. The pain stimuli have been shown to activate aMCC and AI bilaterally, in a pattern similar to that seen in studies where pain has been inflicted to a person present in the same room^9^. The stimulus set is available at https://doi.org/10.6084/m9.figshare.3490421.v1. Participants rated the intensity of unpleasant affect experienced while seeing each stimulus on a visual analogue scale from 0 to 100 by moving a cursor, using a box with 3 buttons (move right, move left and respond) in their right hand. Participants were instructed that 0 would represent no unpleasantness and 100 would represent the worst imaginable unpleasantness. This rating measure has been successfully used before to measure vicarious (shared) affect, a central component of empathy^36^. The cursor was shown at different starting points on every trial to avoid anchoring bias. Stimuli were shown using Presentation software (www.neurobs.com) through eye-tracker goggles (VisualSystem, NeuroLab), adjusted for participants’ visual acuity. Participants’ ratings were recorded using Presentation software. It has been estimated that as many as 30% of subjects may fall asleep during resting state scanning, even during non-sleep deprived conditions^37^, and we therefore verified that no participants fell asleep during the experiment using an eye-tracker, which was also used to record pupil diameter. Heart rate was recorded using a pulse oximeter on the left middle finger.

### Analyses of participants’ ratings

Effects of stimulus type (pain or no pain), sleep condition (full sleep or sleep restriction) and age group (young or older) on rated perceived unpleasantness were investigated using mixed effect models, to handle variance due to repeated measures in the same subjects and slightly unbalanced group sizes. A random intercept was modelled for each subject and stimulus type and sleep condition were entered as fixed within-subjects effects. Age group was modelled as a fixed between-subject effect. All fixed effects were allowed to interact. Results are reported as model estimates in original units (ratings from 0 to 100) with 95% confidence intervals. To control that observed results were not due to possibly confounding variables, test time type (whether participants were scheduled early or late in the evening), session (1st or 2nd scanning), and sex were added sequentially as covariates. Sex and session interacted significantly with stimulus type. In general, unadjusted results are reported. Adjusted results are only shown when differing from unadjusted. Since we did not expect any unpleasantness for the no pain stimuli, introducing a floor effect and unequal variance, the main analyses were performed stratifying for stimulus type.

Possible predictors for empathic responses (self-rated empathy, self-rated psychopathy and self-rated sleepiness) were included as fixed effects one by one in the model. The same procedure was followed for potential habituation effects, where stimulus number was included as a fixed effect. Differences in variability for ratings were investigated using mixed effects models, since we thought that sleep restriction might affect accuracy in responding. Behavioural data were analysed using R version 3.3.0^38^ and scripts can be found at https://doi.org/10.5281/zenodo.846863.

### Analyses of pupil diameter and heart rate

Heart rate recordings were manually inspected for quality and sessions of poor quality were excluded. As described in ref. ^25^, time courses were inspected for each participant and recordings judged as excessively noisy were excluded (*n* = 22, 14%). Heart rates <40 beats per minute (bpm) or >100 bpm were considered non-physiological and were censored. Recordings with less than 50% of data remaining would have been excluded at this stage, but were not present (*n* = 0). Where possible, censored data were imputed using carry-forward of the last non-censored heart rate, which is likely to be a conservative procedure when investigating event-related responses, since it will tend to underestimate changes. 4% of data were censored, out of which 78% were imputed. For each stimulus, heart rate was normalized to the baseline 4 seconds before stimulus onset, and averaged for a window 3.5–5.5 seconds after stimulus onset (poststimulus window). These values were entered into mixed-effects models. The final dataset contained 4578 observations from 115 sessions in 68 subjects.

Pupil diameter recordings were discarded where height or width were <0.1 mm or >0.3 mm, or where the first derivative was <−3 or >3, indicating loss of tracking e.g. due to eye blinks. For events where at least half the data were retained in a window from −4 to 10 seconds after stimulus onset, height and width were interpolated using loess regression. Pupil diameter was calculated as an average of recorded height and width, was normalized to the baseline 4 seconds before stimulus onset, and averaged across time for windows 0–3.5 and 3.5–5.5 seconds after stimulus onset. These values were entered into mixed-effects models. The final dataset contained 2507 observations from 102 sessions in 73 subjects (38% of data retained from all 166 recorded sessions).

### Magnetic resonance image acquisition and preprocessing

Imaging data were acquired using a 3.0 T scanner (Discovery MR750, GE) and an 8-channel head-coil. Foam wedges, earplugs, and headphones were used to reduce head motion and scanner noise. We acquired T1-weighted structural images with whole-head coverage, TR = 6.4 s, TE = 2.8 s, acquisition time 3.58 min and flip angle = 11°, without fat suppression at both sessions. Structural scans were inspected and the one with best quality was used in fMRI data preprocessing. Functional images were acquired using gradient echo-planar-imaging (EPI), TR = 3 s, TE = 34 ms, flip angle = 80°, slice thickness 2.3 mm with 0.1 mm spacing, axial orientation, frequency direction R/L, interleaved bottom up. Higher order shimming was performed and the number of dummy scans before the experiment was 5. The number of slices was 45 or 46. Field of view was placed so that the lowest slice was at the lower margin of the pons. B0 maps were acquired for 40 of the young and 37 of the older participants at both sessions, dTE 2 s. Some additional participants had a B0 map for one of 2 sessions, but to avoid confounding between sessions those were not used.

Imaging data were analysed using SPM12 (http://www.fil.ion.ucl.ac.uk/spm/software/spm12/) running on Matlab2014 (MATLAB 2014, The MathWorks, Inc., Massachusetts, United States). Voxel displacement maps were created from B0 maps using the real and imaginary option in the Fieldmap toolbox. Functional images were preprocessed separately for each session. Images were slice-time corrected with slice no 2 as reference using sinc-interpolation, followed by realignment and unwarping, with 2nd degree B-spline interpolation using a voxel displacement map when possible (see above). Movement parameters were converted to framewise displacement time series, defined as the sum of the absolute values of the derivatives of the six realignment parameters after rotational displacements were converted from degrees to millimetres by calculating displacement on the surface of a sphere of radius 50 mm. Mean framewise displacement did not differ significantly between age groups or sleep conditions and is presented in suppl. Figure 1.

Functional and structural images were co-registered, structural image was set as reference image, mean EPI as source image and all EPI images as “other”. Structural images were segmented and a group specific template was created using DARTEL with default settings in SPM12^39^. Functional images were then normalized to MNI space using flow fields from the previous step through the DARTEL tool. This routine includes an initial affine registration of the DARTEL template with the tissue probability map data released with SPM. Smoothing was performed with a FHWM 8 × 8 × 8 mm kernel. Voxel size after preprocessing was 1.5 × 1.5 × 1.5 mm. All structural images and the first functional image in each session were inspected before, during, and after preprocessing. Scripts used in the preprocessing, as well as in the analyses can be found at https://zenodo.org/record/846863#.WZyEsCig9nI.

### Analysis of fMRI data

Fixed effects models at 1st level included regressors for pain and no pain stimuli, convolved with the canonical hemodynamic response function. Rating events as well as button presses and movement parameters were included as regressors of no interest. Contrasts of interest were pain>no pain, pain>baseline and no pain>baseline, to investigate effects of sleep restriction and age on both pain stimuli and control stimuli.

At 2nd level, one sample t-tests were used to investigate main effects of stimulus type across age groups and sleep conditions. F contrasts were used to test effects of sex, test time type, and session as covariates. At a significance level of *p* < 0.001 uncorrected, none of the covariates showed significant effects in relevant areas on the main contrast pain>no pain, and main analyses were performed without controlling for them.

To investigate effects of sleep condition and age group on 1st level contrasts, we used a flexible factorial design. Sleep condition was entered as a within-subject factor and age group as a between-subject factor. To confirm directions of interaction effects, mean contrast values were extracted from significant clusters and plotted.

To test the hypothesis that sleep restriction would cause less AI and aMCC activity, we defined regions of interest as spheres with a radius of 10 mm around peak coordinates from the meta-analysis by Lamm *et al*.^9^, as done in ref. ^11^. Mean contrast values for pain>no pain in these regions were extracted per subject and session and entered in mixed effects models. To test associations between activity in AI and aMCC and IRI-EC and PPI-R, these variables were separately added to the models.

To test the association between rated unpleasantness and fMRI responses an alternative 1st level model was created, in which participants’ ratings of perceived unpleasantness were entered as parametric modulators for pain, and brought to 2nd level for a 1 sample t test. Statistical maps from second level analysis can be found at https://neurovault.org/collections/1219/. When not stated differently, *p*_*FWE*_ < 0.05 at peak level was considered significant. For analyses including sleep and age, results are reported at *p* < 0.001 unc. in tables and figures for illustrative purposes. Cluster corrected statistics are provided in supplement.

### Data availability

The datasets analysed in the current study are available at https://doi.org/10.5281/zenodo.846863 and in the OpenfMRI repository at https://openfmri.org/dataset/ds000201/.

## Results

### Characteristics of participants

Descriptive statistics of rating scales, sleep measures and demographic data are presented in Table 1. KSS ratings showed increased sleepiness after sleep restriction, reported as effect estimates in original units with 95% CI (1.6 [2.2, 1.1], *t*(169), *p* < 0.001, Table 1). Ratings of sleepiness and sleep measures are reported in detail in Åkerstedt *et al*. (submitted).

**Table 1:** Demographic data. Continuous values are reported as means with standard deviations, unless otherwise indicated.

Variables	Young	Old
**Sample**		
Number of subjects	47	39
**Demographics**		
Age (median, interquartile range)	23.0 (21.5–25.0)	68.0 (67.0–71.0)
Sex (females)	24 (51.1%)	20 (51.3%)
Body Mass Index	22.9 (±3.1)	24.7 (±3.4)
**Education**		
Elementary school	1 (2.1%)	3 (7.7%)
High school	10 (21.3%)	17 (43.6%)
University degree	6 (12.8%)	18 (46.2%)
University student	30 (63.8%)	1 (2.6%)
**Interpersonal reactivity index**		
Empathic concern	3.9 (±0.6)	3.9 (±0.4)
Fantasy	3.4 (±0.7)	2.9 (±0.6)
Perspective taking	3.6 (±0.6)	3.8 (±0.4)
Personal distress	2.6 (±0.6)	2.4 (±0.7)
**HADS**		
Depression	1.1 (±1.4)	1.2 (±1.0)
Anxiety	2.8 (±2.4)	1.4 (±1.4)
**Sleep**		
Insomnia severity index	10.6 (±2.1)	9.3 (±1.7)
Karolinska Sleepiness Scale, full sleep	5.9 (±1.8)	4.5 (±1.8)
Karolinska Sleepiness Scale, sleep restriction	7.7 (±1.4)	5.8 (±1.7)
Total sleep time (min), full sleep	429.1 (±77.4)	388.9 (±68.3)
Total sleep time (min), sleep restriction	185.3 (±36.7)	158.9 (±31.9)
REM sleep (min), full sleep	86.8 (±29.9)	74.9 (±35.6)
REM sleep (min), sleep restriction	28.2 (±15.8)	25.7 (±18.7)
Slow wave sleep (min), full sleep	98.0 (±32.0)	41.1 (±33.1)
Slow wave sleep (min), sleep restriction	70.5 (±16.5)	27.2 (±24.4)

Categorical data are reported with percentages. Sleep measures are reported in minutes.

### Ratings of unpleasantness

#### Overall model

**Figure 3:**
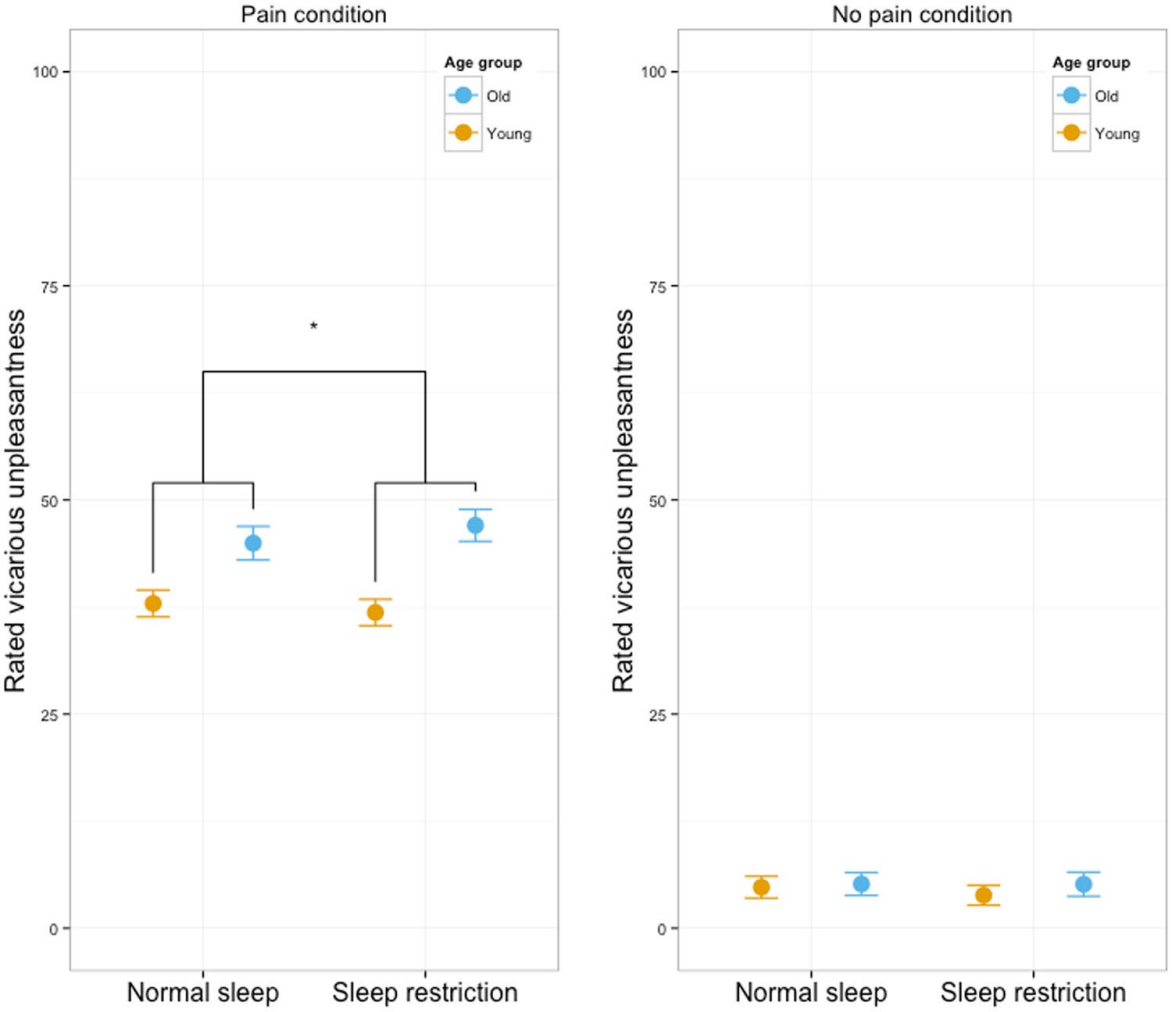
Ratings of perceived unpleasantness. Means and 95% CI.

Results are reported as effect estimates (rated unpleasantness) in original units with 95% CI. In an overall model, the three-way interaction stimulus type*sleep condition*age group was not significant (2.24 [−1.52, 5.99], *p* = 0.24). Effects of stimulus type (36.95 [36.01, 37.89], *p* < 0.0001) and stimulus type*age group (7.79 [5.91, 9.66], *p* < 0.0001) were strong. All other effects were non-significant. Results are shown in Fig. 3.

#### Pain stimuli caused increased unpleasantness compared to no pain stimuli

Pain stimuli were rated as causing a more unpleasant experience than no pain stimuli, both for young (32.5 [31.4, 33.7], *p* < 0.001, Fig. 3) and older (38.8 [37.4, 40.1], *p* < 0.001, Fig. 3) participants, confirming expected vicarious responding. There was a small increase in rated unpleasantness for pain stimuli over the time-course of the scanning session (3.7 [1.8, 5.6], *p* < 0.001), but not for no pain stimuli (0.6 [−0.4, 1.7], *p* = 0.2). For no pain stimuli, participants generally did not report any unpleasantness (median 0, interquartile range 0–2).

#### Sleep restriction caused increased unpleasantness in older, but not younger, participants

For pain stimuli, age group interacted with sleep condition (3.1 [0.8, 5.5], *p* = 0.01, Fig. 3). When decomposed by age group, older participants reported increased unpleasantness (2.1 [0.2, 4.0], *p* = 0.03, Fig. 3), whereas no significant effect was seen in young participants (−1.1 [−2.5, 0.4], *p* = 0.16, Fig. 3) after sleep restriction. Across age groups, the effect of sleep restriction on unpleasantness in response to pain stimuli was not significant (0.5 [−0.7, 1.7], *p* = 0.4, Fig. 3). As reported above, age group and stimulus type interacted significantly in the full model. Across sleep conditions, older participants reported more unpleasantness to the pain stimuli, compared to young, but the difference was not significant (6.2 [−5.3, 17.7], *p* = 0.29, Fig. 3), hence the significant age group*stimulus type interaction. There was no significant main effect of age group on no pain stimuli (0.5 [−3.4, 4.5], *p* = 0.78, Fig. 3), nor any age group*sleep condition interaction (0.9 [−0.3, 2,2], *p* = 0.2). There was no effect of sleep restriction on perceived unpleasantness to no pain stimuli across age groups (−0.5 [−1.1, 0.1], *p* = 0.15, Fig. 3).

Sleep restriction did not cause any change in variability in ratings for pain (−0.2 [−1.9, 1.5], *p* = 0.81) or no pain (1.2 [−0.4, 2.8], *p* = 0.16). Variability in ratings did not differ between age groups, neither for pain (−0.7 [−3.5, 2.1], *p* = 0.60), nor no pain (−0.1 [−2.6, 2.4], *p* = 0.93).

#### Trait empathy predicted ratings of unpleasantness

In an unadjusted model, self-rated empathy (IRI-EC) interacted with stimulus type (6.6 [4.8, 8.4], *p* < 0.0001). However, no association between rated unpleasantness and IRI-EC was seen in pain (6.6 [−4.9, 18.0], *p* = 0.3, suppl. Figure 5) or no pain (−0.2 [−4.2, 3.7], *p* = 0.9, suppl. Figure 5) when analysing the conditions separately. When including sex in the full model, the interaction was no longer significant (−0.9 [−2.9, 1.1], *p* = 0.37). Self-rated psychopathy (PPIR, coldheartedness) interacted with stimulus type (−1.1 [−1.27, −0.96], *p* < 0.0001). No association between rated unpleasantness and coldheartedness was seen in pain (−0.9 [−1.9, 0.11], *p* = 0.08) or no pain (0.23 [−0.12, 0.59], *p* = 0.20).

### Heart rate and pupil diameter

#### Sleep restriction was associated with increased pupil constriction

Inspection of pupil diameter responses confirmed that stimulus onset was followed by constriction, as expected due to more light reaching the retina (suppl. Figure 2a and b). Pain stimuli displayed on inspection a time-course with less constriction (suppl. Figure 2a) and sleep restriction a time-course with more constriction (suppl. Figure 2b). When diameter change was averaged over events and entered into a regression model with the tree-way interaction between stimulus type, restriction condition, and age group, only the effect of pain vs no pain was statistically significant (0.004 mm [0.002, 0.005], *p* < 0.001, suppl. Figure 2c), indicating that pain stimuli were associated with less constriction. In a reduced model not including age group, the effect of sleep restriction was −0.003 mm [−0.006, −0.000], *p* = 0.04], indicating that sleep restriction was associated with more constriction.

#### No effect of sleep restriction on heart rate

Inspection of heart rate responses confirmed that stimulus onset was followed by deceleration, as expected due to processing of emotionally salient stimuli (suppl. Figure 3a and b). Pain stimuli displayed on inspection a time-course with less deceleration (suppl. Figure 3a) and sleep restriction a time-course with more deceleration (suppl. Figure 3b). When heart rate was averaged over events and entered into a regression model with the three-way interaction between stimulus type, restriction condition, and age group, no effects were statistically significant (suppl. Figure 3c).

### fMRI results

#### Pictures of pain in others activated bilateral AI and aMCC

**Figure 4:**
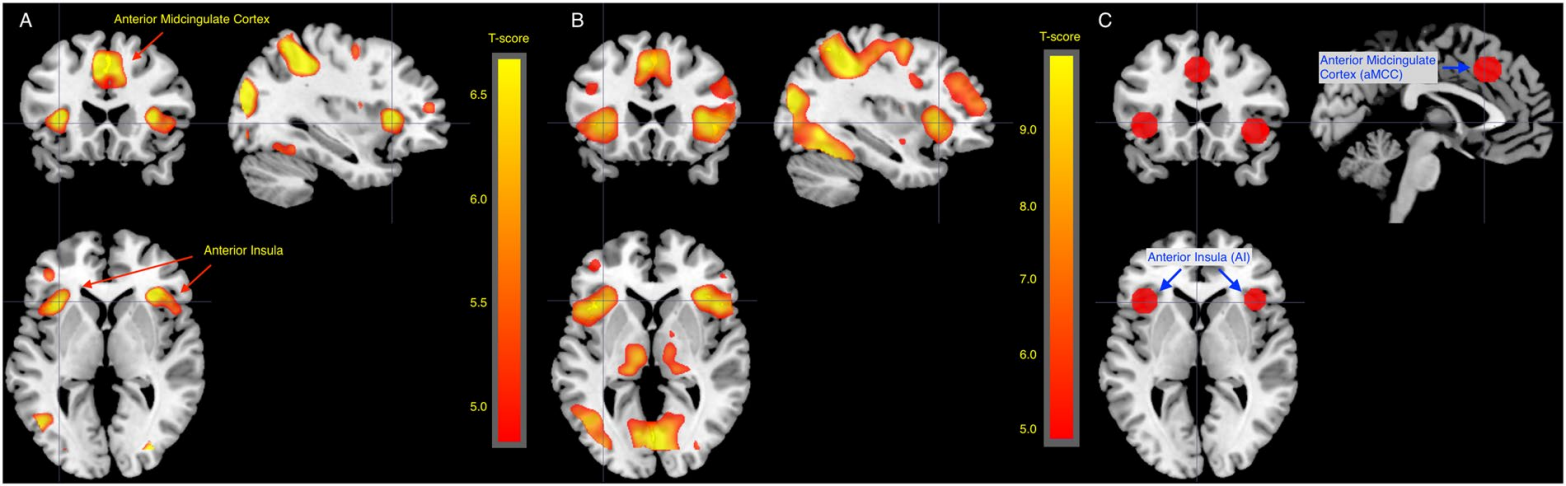
Figures showing main effects of stimulus type across all subjects and sleep conditions. (**A**) Pain>no pain across all subjects and sleep conditions, thresholded at p < 0.05 FWE for the whole brain. Activity was observed in AI and aMCC. (**B**) Pain>baseline across all subjects, p < 0.05 FWE. Activation maps include AI and aMCC, but larger areas. (**C**) Regions of interest in AI (−40, 22, 0 and 39, 23, −4) and aMCC (−2, 23, 40) based on coordinates from the meta-analysis by Lamm *et al*. with 10 mm spheres around each coordinate.

In the contrast pain>no pain, increased activity was observed in bilateral AI and aMCC (Table 2, Fig. 4a). Moreover, increased activity was observed in parietal and occipital areas, around the frontal gyri and in the thalamus. These activations overlaps with results of a meta-analysis of empathy for pain studies^9^. As both sleep restriction and age group might affect responses to control stimuli, confounding other analyses, we also investigated the contrast pain>baseline (fixation cross and blank screen). This contrast resulted in activity similar to the contrast pain>no pain, but showing larger clusters (Fig. 4b, Table 3

**Table 2:** Pain>no pain for all subjects across sleep conditions, thresholded at 0.05 FWE corrected and only clusters with >20 voxels are reported.

Anatomical area (peak coordinate)	MNI coords mm (x, y, z)	cluster	peak	peak	peak	peak
MRIcron (automated anatomical labeling)		equivk	p(FWE-corr)	T	equivZ	p(unc)
Supramarginal (L)	−58, −27, 34	12036	<0.001	11.93	Inf	<0.001
Superior parietal (L)	−16, −68, 54		<0.001	9.44	7.78	<0.001
Inferior parietal (L)	−33, −46, 50		<0.001	9.02	7.53	<0.001
Inferior occipital (R)	33, −86, −4	6294	<0.001	10.15	Inf	<0.001
Middle occipital (R)	38, −82, 10		<0.001	8.78	7.39	<0.001
Middle occipital (R)	38, −74, 28		<0.001	8.32	7.1	<0.001
Precentral (L)	−51, 6, 30	1599	<0.001	9.31	7.71	<0.001
Rolandic operculum (L)	−52, 6, 9		0.006	5.43	5.02	<0.001
Superior medial frontal (L)	−4, 21, 44	3203	<0.001	8.75	7.37	<0.001
Middle cingulate cortex (L)	−4, 27, 38		<0.001	8.39	7.14	<0.001
Anterior cingulate cortex (L)	−2, 18, 27		0.029	5	4.67	<0.001
Middle frontal (L)	−26, 0, 52	1141	<0.001	8	6.89	<0.001
Insula (R)	32, 24, 4	2101	<0.001	7.69	6.68	<0.001
Inferior frontal operculum (R)	51, 9, 27		<0.001	7.41	6.49	<0.001
Inferior frontal operculum (R)	50, 15, 4		0.003	5.65	5.19	<0.001
Insula (L)	−34, 20, 6	1054	<0.001	7.52	6.56	<0.001
Inferior frontal, triangular part (L)	−44, 39, 6	1034	<0.001	6.6	5.92	<0.001
Inferior frontal, triangular part (L)	−42, 33, 27		<0.001	6.16	5.59	<0.001
Middle frontal (L)	−30, 48, 14		0.001	5.84	5.34	<0.001
Precentral (R)	30, −2, 52	526	<0.001	6.36	5.73	<0.001
Insula (L)	−36, −2, 15	43	0.002	5.79	5.3	<0.001
NA	−8, −24, −12	114	0.003	5.61	5.16	<0.001
Thalamus (L)	−12, −15, 9	45	0.006	5.43	5.02	<0.001

Anatomic labels are shown for peak coordinates and were defined using the automatic anatomic labelling in MRIcron.

).

**Table 3:** Pain>baseline for all subjects across sleep conditions, thresholded at 0.05 FWE corrected and only clusters with >20 voxels are reported.

Anatomical area (peak coordinate)	MNI coords mm (x, y, z)	cluster	peak	peak	peak	peak
MRIcron (automated anatomical labeling)		equivk	p(FWE-corr)	T	equivZ	p(unc)
Inferior parietal (L)	−30, −46, 45	51828	<0.001	13.92	Inf	<0.001
Middle occipital (R)	33, −80, 32		<0.001	13.59	Inf	<0.001
Superior parietal (L)	−20, −66, 51		<0.001	13.05	Inf	<0.001
Inferior frontal operculum (R)	45, 9, 28	9990	<0.001	12.16	Inf	<0.001
Insula (R)	34, 24, 4		<0.001	10.57	Inf	<0.001
Precentral (R)	38, 2, 52		<0.001	10.19	Inf	<0.001
Supplemental motor area (L)	−6, 9, 50	4590	<0.001	12.09	Inf	<0.001
Supplemental motor area (R)	8, 14, 51		<0.001	11.29	Inf	<0.001
Anterior cingulate cortex (R)	8, 15, 28		0.001	5.85	5.35	<0.001
NA	−8, −20, −8	3950	<0.001	10.09	Inf	<0.001
Thalamus (L)	−14, −21, 6		<0.001	8.8	7.4	<0.001
Thalamus (L)	−16, −28, 2		<0.001	8.5	7.21	<0.001
Middle frontal (L)	−42, 34, 32	3801	<0.001	7.95	6.85	<0.001
Middle frontal (L)	−32, 51, 14		<0.001	7.47	6.53	<0.001
Middle frontal (L)	−40, 40, 22		<0.001	7.38	6.47	<0.001
NA	14, 0, 2	25	0.004	5.62	5.17	<0.001
NA	−34, −3, −12	20	0.011	5.31	4.92	<0.001

Anatomic labels are shown for peak coordinates and were defined using the automatic anatomic labelling in MRIcron.

#### Sleep restriction did not affect BOLD responses to pain in others

No voxels showed a main effect of sleep condition, neither for the contrast pain>no pain nor for pain>baseline (*p* < 0.05 FWE). With a more liberal threshold (*p* < 0.001 unc), full sleep compared to sleep restriction (pain>no pain) yielded increased activity in 4 small voxel clusters that were judged as random findings, because of their small sizes (available at https://doi.org/10.5281/zenodo.846863). To test the specific hypothesis that sleep restriction inhibits AI and aMCC responses, a region of interest analysis was performed for the contrast pain>no pain, see Fig. 4c for ROIs. In the ROI analysis, we found no effect of sleep restriction in AI (right: (−0.12 [−0.45, 0.20], *p* = 0.45, left: (0.03 [−0.31, 0.37], *p* = 0.88) nor aMCC (0.05 [−0.26, 0.34], *p* = 0.78), results presented as parameter estimates in original units (contrast values) with 95% CI.

### Older participants showed more activity in the angular gyrus

**Figure 5:**
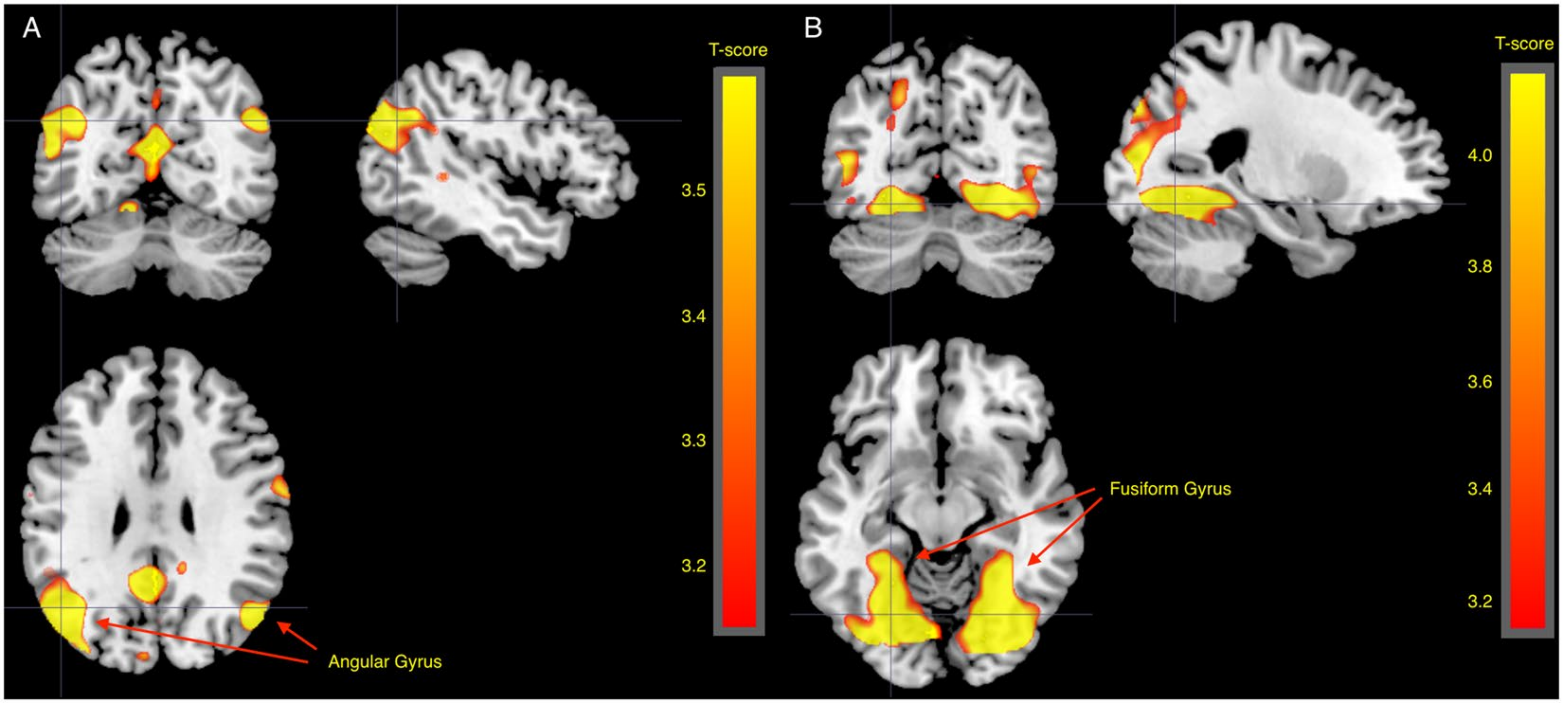
Age effects. (**A**) Older>young for the contrast pain>no pain, 0.001 uncorrected for illustration. Older participants showed more activity in bilateral angular gyrus compared to young participants. (**B**) Older>young for the contrast pain>baseline, 0.001 uncorrected for illustration. Older participants showed more activity in bilateral fusiform gyrus compared to young participants.

Pain>no pain in older participants compared to young yielded increased activity in the angular gyrus bilaterally, superior temporal sulcus (STS) and temporo-parietal junction (TPJ) and around the calcarine fissure (*p* < 0.05 FWE, Fig. 5a, Table 4a). Both pain and no pain stimuli compared to implicit baseline caused increased activity in the fusiform gyrus in older compared to younger participants (*p* < 0.05 FWE, Table 4c, Fig. 5b and suppl. Figure 4 and suppl. Table 1).

**Table 4:** Effects of age. All tables show data thresholded at 0.001 uncorrected, only clusters with>20 voxels are shown.

A						
Anatomical area (peak coordinate)	MNI coords mm (x, y, z)	cluster	peak	peak	peak	peak
MRIcron (automated anatomical labeling)		equivk	p(FWE-corr)	T	equivZ	p(unc)
Angular (L)	−48, −74, 30	2857	<0.001	6.4	5.98	<0.001
Middle occipital (L)	−40, −78, 36		0.001	5.8	5.48	<0.001
Angular (L)	−40, −62, 32		0.143	4.33	4.19	<0.001
Calcarine (L)	−2, −87, 14	9176	0.005	5.27	5.02	<0.001
Cuneus (L)	−10, −93, 14		0.007	5.18	4.94	<0.001
Calcarine (L)	−2, −63, 16		0.017	4.95	4.74	<0.001
Angular (R)	48, −69, 32	589	0.025	4.84	4.65	<0.001
Superior frontal (R)	30, 27, 52	860	0.028	4.81	4.62	<0.001
Middle frontal (R)	26, 42, 42		0.327	4.04	3.92	<0.001
Middle temporal (L)	−56, −48, −6	1025	0.078	4.52	4.36	<0.001
Middle temporal (L)	−51, −42, 0		0.672	3.7	3.6	<0.001
Middle temporal (L)	−56, −36, 10		0.891	3.44	3.37	<0.001
Middle frontal (L)	−27, 38, 45	250	0.256	4.13	4.01	<0.001
Superior frontal (L)	−21, 44, 44		0.745	3.62	3.53	<0.001
Superior frontal (L)	−22, 36, 36		0.958	3.3	3.23	0.001
Superior temporal (R)	64, −9, 8	311	0.457	3.9	3.79	<0.001
Rolandic operculum (R)	51, −8, 20		0.957	3.3	3.23	0.001
Postcentral (R)	64, −6, 27	254	0.644	3.72	3.63	<0.001
Precentral (R)	20, −22, 66	54	0.653	3.71	3.62	<0.001
Precentral (R)	30, −22, 64		0.939	3.35	3.28	0.001
Medial frontal orbital (R)	2, 54, −8	111	0.676	3.69	3.6	<0.001
Cerebelum (R)	12, −54, −8	134	0.707	3.66	3.57	<0.001
Precentral (R)	45, −10, 52	193	0.715	3.65	3.56	<0.001
Parahippocampal (L)	−20, −38, −8	194	0.717	3.65	3.56	<0.001
Parahippocampal (L)	−27, −36, −10		0.791	3.57	3.49	<0.001
Superior temporal (L)	−54, −15, 2	26	0.732	3.64	3.55	<0.001
Superior temporal (R)	46, −32, 15	77	0.784	3.58	3.5	<0.001
Superior temporal (R)	57, −2, −8	23	0.869	3.48	3.4	<0.001
Precentral (R)	39, −15, 38	28	0.966	3.28	3.21	0.001
Precentral (R)	36, −15, 48		0.989	3.16	3.1	0.001
**B**						
**Anatomical area (peak coordinate)**	**MNI coords mm (x, y, z)**	**cluster**	**peak**	**peak**	**peak**	**peak**
MRIcron (automated anatomical labeling)		equivk	p(FWE-corr)	T	equivZ	p(unc)
Anterior cingulate cortex (R)	9, 24, 26	122	0.334	4.15	4.03	<0.001
Postcentral (L)	−39, 22, 50	98	0.604	3.89	3.78	<0.001
Postcentral (L)	−51, −26, 57	25	0.984	3.34	3.27	0.001
**C**						
**Anatomical area (peak coordinate)**	**MNI coords mm (x, y, z)**	**cluster**	**peak**	**peak**	**peak**	**peak**
MRIcron (automated anatomical labeling)		equivk	p(FWE-corr)	T	equivZ	p(unc)
Fusiform (L)	−24, −75, −10	15740	<0.001	7.93	7.19	<0.001
Fusiform (L)	−28, −60, −10		<0.001	7.56	6.9	<0.001
Fusiform (R)	28, −66, −9		<0.001	7.28	6.69	<0.001
Precentral (R)	58, 2, 40	311	0.081	4.62	4.45	<0.001
Precentral (R)	63, 9, 26		0.933	3.51	3.43	<0.001
Superior occipital (R)	24, −81, 40	348	0.083	4.61	4.44	<0.001
Superior occipital (R)	32, −76, 45		0.871	3.61	3.53	<0.001
Superior parietal (R)	34, −45, 62	1463	0.116	4.51	4.35	<0.001
Inferior parietal (R)	28, −48, 54		0.183	4.37	4.22	<0.001
Postcentral (R)	32, −33, 51		0.802	3.7	3.6	<0.001
Middle frontal (R)	44, −3, 54	817	0.12	4.5	4.34	<0.001
Precentral (R)	34, −2, 50		0.334	4.15	4.03	<0.001
Superior frontal (R)	32, −4. 62		0.693	3.81	3.71	<0.001
Postcentral (L)	−62, −15, 30	799	0.282	4.22	4.08	<0.001
Supramarginal (L)	−58, −26, 26		0.298	4.2	4.07	<0.001
Postcentral (L)	−51, −22, 33		0.903	3.57	3.48	<0.001
Calcarine (R)	21, −54, 10	360	0.304	4.19	4.06	<0.001
Lingual (R)	6, −60, 9		0.955	3.46	3.38	<0.001
NA	9, −27, −4	472	0.479	4	3.89	<0.001
NA	−3, −45, 8		0.857	3.63	3.54	<0.001
NA	−6, −28, −8		0.906	3.56	3.48	<0.001
Superior parietal (L)	−33, −57, 63	39	0.707	3.79	3.69	<0.001
Inferior parietal (L)	−34, −40, 48	63	0.806	3.69	3.6	<0.001
NA	24, −26, −4	32	0.852	3.64	3.55	<0.001
Inferior frontal operculum (L)	−44, 6, 30	248	0.866	3.62	3.53	<0.001
Precentral (L)	−50, 3, 34		0.868	3.62	3.53	<0.001
Postcentral (R)	66, −12, 22	31	0.884	3.59	3.51	<0.001
Inferior frontal operculum (L)	−52, 9, 20	109	0.914	3.55	3.46	<0.001
Precentral (L)	−44, −3, 58	75	0.932	3.51	3.43	<0.001
Precentral (L)	−40, −4, 51		0.975	3.39	3.32	<0.001
Superior temporal (L)	−46, −8, −6	36	0.95	3.47	3.39	<0.001
Middle cingulate cortex (R)	12, −20, 44	25	0.961	3.44	3.36	<0.001
Postcentral (R)	60, −12, 40	86	0.967	3.42	3.35	<0.001
Posterior cingulate cortex (L)	−8, −36, 34	26	0.971	3.41	3.33	<0.001
Middle frontal (R)	33, 38, 40	30	0.979	3.37	3.3	<0.001

Anatomic labels are shown for peak coordinates and were defined using the automatic anatomic labelling in MRIcron. A. The contrast older>young for pain>no pain, B. The contrast young>older for pain>baseline. C. The contrast older>young for pain>baseline.

**Figure 6:**
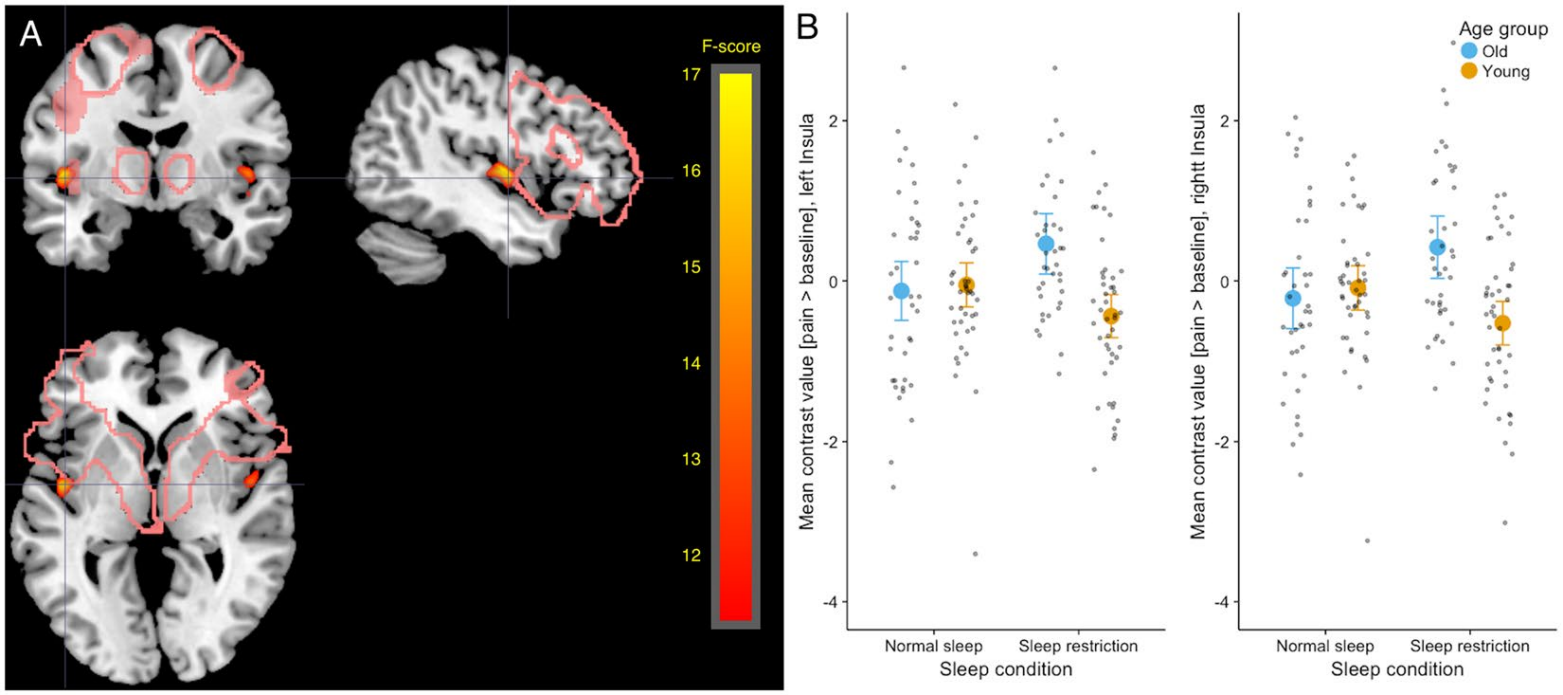
Showing age x sleep interaction for the contrast pain>baseline in a cluster in bilateral insula. After sleep restriction, older participants showed increased activity in pain>baseline compared to normal sleep, whereas younger participants showed decreased activity for the same contrast after sleep restriction compared to normal sleep. (**A**) Sleep x age interaction on pain>baseline, thresholded at 0.001 uncorrected. Pink lines indicate areas known as the empathy network from the meta-analysis by Lamm *et al*. (**B**) Mean contrast values for pain>baseline extracted from clusters in insula per subject and session and plotted per age group and condition. No post hoc test was performed on these extracted values, since they were extracted based on the interaction and thus would result in “double-dipping”, inflating results.

With a threshold of p < 0.05 FWE, age group did not interact with sleep condition. However, with a more liberal threshold at p < 0.001 unc., clusters on the border between anterior and posterior insula showed an interaction effect for the contrast pain>baseline (*p* < 0.001 unc., Fig. 6a and b, Table 5), consistent bilaterally. In these clusters, older participants showed more activity in the contrast pain>baseline after sleep restriction, whereas young participants showed decreased activity after sleep restriction.

**Table 5:** Table showing the interaction contrast age group*sleep condition for pain>baseline, thresholded at 0.001 uncorrected, only clusters with>20 voxels are shown.

Anatomical area (peak coordinate)	MNI coors mm (x, y, z)	cluster	peak	peak	peak	peak
MRIcron (automated anatomical labeling)		equivk	p(FWE-corr)	F	equivZ	p(unc)
Insula (L)	−45, −8, 3	260	0.624	16.79	3.8	<0.001
Insula (R)	46, −4, 2	159	0.929	14.2	3.49	<0.001
Superior temporal (R)	46, −3, −8		0.95	13.88	3.45	<0.001

Anatomic labels are shown for peak coordinates and were defined using the automatic anatomic labelling in MRIcron.

#### Neither rated unpleasantness nor trait empathy predicted fMRI responses

No voxels showed an effect of rated unpleasantness to stimuli. Self-rated empathy (IRI-EC) did not predict responses in AI (right: (−0.09 [−0.29, 0.17], *p* = 0.38, left: (−0.14 [−0.34, 0.07], *p* = 0.19)) or aMCC (−0.04 [−0.27, 0.18], *p* = 0.57) for pain>no pain, results presented as parameter estimates in original units (contrast values) with 95% CI. Self-rated psychopathy (PPI-R, coldheartedness) did not significantly predict responses in AI (right: (0.01 [−0.01, 0.02], *p* = 0.46, left: (0.01 [−0.00, 0.03], *p* = 0.15)) or aMCC (0.01 [−0.01, 0.03], *p* = 0.39) for pain>no pain.

## Discussion

This study investigated the effect of sleep restriction on empathic responses to pain in older and younger participants. Across age groups, there was no significant effect of sleep restriction on empathic responding. Older participants had higher activity in angular gyrus, STS, TPJ and calcarine fissure in response to pain, contrasted to no pain, in others. Age group and sleep interacted so that sleep restriction caused increased unpleasantness to pain in older participants and no effect in young. In voxel clusters in bilateral posterior insula, sleep restriction tended to cause increased activity in older and decreased activity in young participants, when pain stimuli were contrasted to a baseline, but not when contrasted to no pain stimuli. Behavioural and imaging results could therefore indicate that aging affects both how we respond to pain in others and possibly how sleep modulates emotional responses.

Most studies of sleep and emotion have been performed with young participants, in spite of the fact that normal sleep and the effects of sleep restriction change with aging^4,18^. At an uncorrected threshold, sleep and age group interacted in clusters in posterior insula. The clusters were large and bilateral, but only partly overlapped with the core empathy network observed in previous studies of empathy, and the effects did not survive a whole-brain FWE correction, limiting interpretation. For ratings of unpleasantness, a significant interaction between age group and sleep condition was seen. However, when decomposed, the effect was only significant in the older participants. Although both behavioural and fMRI effects were ambiguous, they are consistent with the interpretation that sleep deprivation affects younger and older participants differently, which would call for caution in generalization across age groups in this area of investigation. Whether potential differences in how younger and older participants respond to sleep restriction depend on the microstructure of the sleep itself, or the function of sleep in recovery needs further attention.

Most effects of sleep restriction on emotional processing in the brain have been investigated in relation to changes in amygdala reactivity^3,8,24,40^, while empathic computations rely more on structures in AI and aMCC^9–11^. Only one previous study^15^ reported decreased self-reported empathy after sleep restriction in young volunteers (mean age = 22), inconsistent with the results of the present study. Regarding emotional recovery, REM sleep has been argued to be of certain importance^41,42^. In the present study, we did not completely abrogate this sleep phase, potentially still allowing some recovery during the experimental night. Whether the brain network underlying empathy is relatively resistant to sleep loss, or whether an impact of sleep deprivation on empathy would arise with a stronger manipulation that e.g. completely eliminates REM, needs to be further studied.

An important aim of the study was to investigate the effect of age on responses to pain in others, and the results indicate greater behavioural responses in older age. This is contrary to what was found by Chen *et al*.^23^, who investigated empathy for pain in three different adult age groups. In addition, unlike Chen *et al*., we found no effects of aging on empathic responses in AI or aMCC. As noted above, older participants however showed increased activity in an area including angular gyrus, TPJ and STS bilaterally, as well as around the calcarine gyrus in response to pain as compared to no pain stimuli. The clusters show some overlap with a proposed theory of mind network^43,44^ and could in speculation indicate an increased recruitment of areas associated with cognitive^43,44^ aspects of empathy (i.e. theory of mind) in older participants when exposed to others in pain, even though this interpretation needs to be tested. Another possible explanation is that older participants are less used to the type of stimuli used, and that this activity represents increased perceptual vigilance. It could partly also represent compensatory recruitment of brain areas in the older group^45^. Older participants have on average less grey matter^46^, possibly affecting the shape and amplitude of the BOLD signal. Since the BOLD signal generally decreases with aging^47^, we suggest that increased BOLD signal in our older participants could potentially reflect an even larger difference in underlying neural activity.

Pain stimuli caused greater BOLD responses in the AI and aMCC than control stimuli, thus validating the paradigm in which the effects of sleep and age on empathy was studied^25^. However, activity in AI and aMCC was not related to ratings of trait empathy or to ratings of unpleasantness during the experiment. This has in fact been the case in most earlier studies of empathy^9^, contrary to an early report of the opposite^10^.

### Strengths and limitations

Many studies of sleep and emotion use total sleep deprivation with stronger assumed effects on behaviour and brain function. Partial sleep restriction is however a more ecologically valid condition, and the use of this protocol therefore increases generalizability. Another disadvantage with total sleep deprivation is that it increases the risk of participants falling asleep in the scanner. In fact, this is implicated as a major confounder in fMRI studies^37^, and the present protocol was piloted to balance sleepiness with the risk to fall asleep during imaging. Our data show that reducing sleep to 3 hours induced considerable sleepiness, which shows that the manipulation was successful. Another strength of the study was the fact that participants were monitored with polysomnography at home. Thus, the participants’ actual sleep was carefully checked, which to our knowledge has not been the case in many sleep restriction studies. Another critical issue in imaging research is statistical power^48^. The sample size in this study is larger than in any other study of effects of sleep manipulations on emotional outcomes, using fMRI, of which we are aware. Still, as the effects of interest may be weak, power may still be an issue. This would especially apply to interactions, where power is further diluted. On the other hand, we pre-registered a list of hypotheses in an attempt to restrict the analyses, reducing the risk of confusing exploratory and confirmatory data analysis. We investigated age effects in a cross-sectional manner, which introduces a risk that the observed effects are not caused by aging itself, but rather by a generational effect (of for example history of pain in self or others, attitudes to pain or exposure to violent pictures). Also, a “healthy volunteer effect”^49^ can be expected in the present design, introducing a selection bias in the older sample. On the other hand, the present sample displays low risk for other confounders such as morbidity related to aging, in other words isolating the effects of age in this respect.

## Conclusions

Despite strong effects on sleepiness, shortened sleep did not have a uniform effect on empathic responding across age groups. In older participants, sleep restriction caused increased perceived unpleasantness to pain in others, whereas no effect was seen in young. No effect of sleep restriction was found in the core empathy network (AI and aMCC). Compared to younger, older participants showed more activity in additional brain areas, including angular gyri, STS and TPJ, in response to pain in others. The pattern of results suggests a complexity in the way age interacts with empathic processing and possibly also with effects of sleep restriction on empathy. This is likely relevant also for the effect of sleep on other domains of emotional processing and calls for caution in generalizability of findings across age groups. It is also necessary to study how sleep-emotion effects translate to prosocial behaviour, and whether this has any implications for health care professions, patients with disturbed sleep or the general population.

## Electronic supplementary material

Supplementary information 
