# Supplementary material for: The effect of sleep restriction on empathy for pain: An fMRI study in younger and older adults
Source: Sci Rep. 2017 Sep 25;7:12236. doi: 10.1038/s41598-017-12098-9 (PMC5612991; doi:10.1038/s41598-017-12098-9)
Supplement: Supplementary file 1 — Supplementary information [file 41598_2017_12098_MOESM1_ESM.pdf]

The effect of sleep restriction on empathy for pain: An fMRI study in younger and older adults

Sandra Tamm, Gustav Nilsonne, Johanna Schwarz, Claus Lamm, Göran Kecklund, Predrag Petrovic, Håkan Fischer, Torbjörn Åkerstedt, Mats Lekander

Supplementary materials

List of specific hypotheses

A complete list of preregistered hypotheses can be found at Open Science Framework (https://osf.io/bxfsb/), where the present experiment is referred to as "HANDS". The hypotheses addressed in this manuscript are listed below. Other hypotheses in the preregistration will be addressed in coming manuscripts.

Self-rated unpleasantness

1: Partial sleep deprivation (PSD) will cause decreased ratings of unpleasantness.

2: Ratings of unpleasantness will be predicted by participants' ratings on the Interpersonal Reactivity Index Empathic Concern subscale (IRI-EC) and inversely predicted by the Psychopathic Personality Index-Revised Coldheartedness subscale (PPI-R-C).

3: Ratings of unpleasantness will habituate.

Pupillometry

1: Pictures with pain stimuli will cause greater pupil dilation than control pictures.

2: PSD will cause lesser pupil dilation in response to pain stimuli.

Heart rate

1: Pain stimuli will cause greater heart rate increases than no pain stimuli and PSD will interact with this increase to cause lesser increases to pain

BOLD response

1: Pain stimuli will cause greater BOLD responses in the anterior insula (AI) and anterior/middle cingulate cortex (ACC/MCC) than control stimuli and PSD will interact with this increase to cause lesser increases to pain stimuli.

2: AI and ACC/MCC BOLD responses will be predicted by IRI-EC ratings and inversely predicted by PPI-R-C ratings (see above).

3: AI and ACC/MCC BOLD responses will be predicted by participants' rated unpleasantness.

Supplementary figures

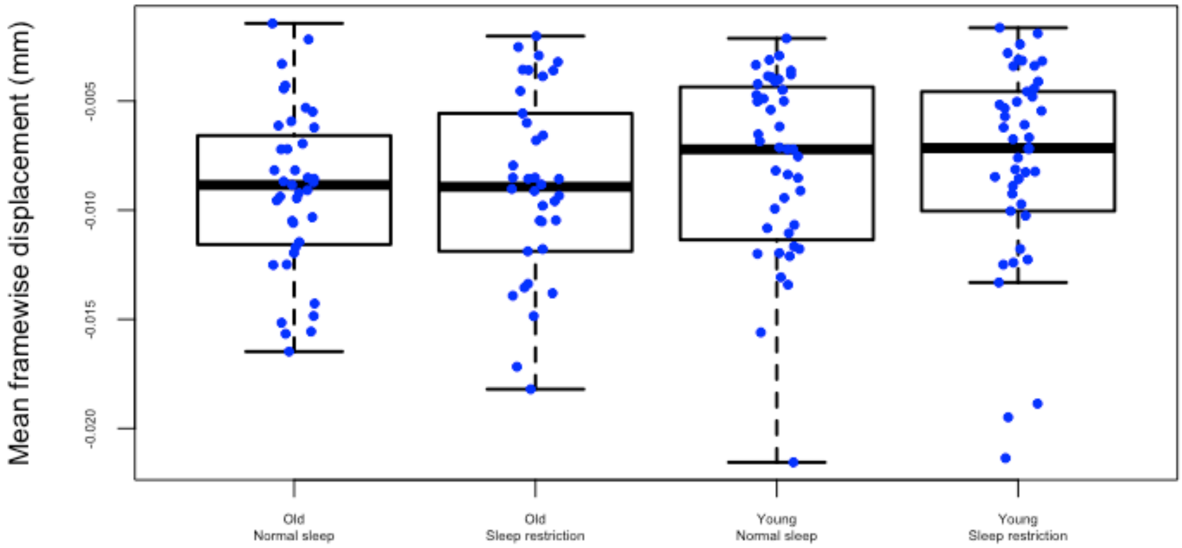

Supplementary figure 1a. Mean framewise displacement plotted per age group and sleep condition, dots show individual participants

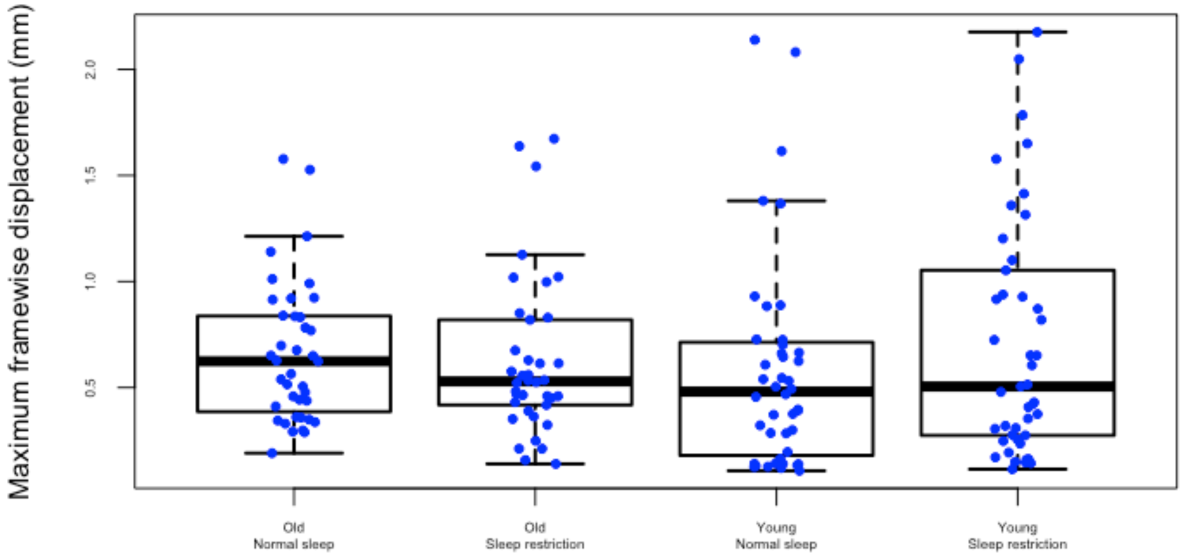

Supplementary figure 1b. Maximum framewise displacement plotted per age group and sleep condition, dots show individual participants.

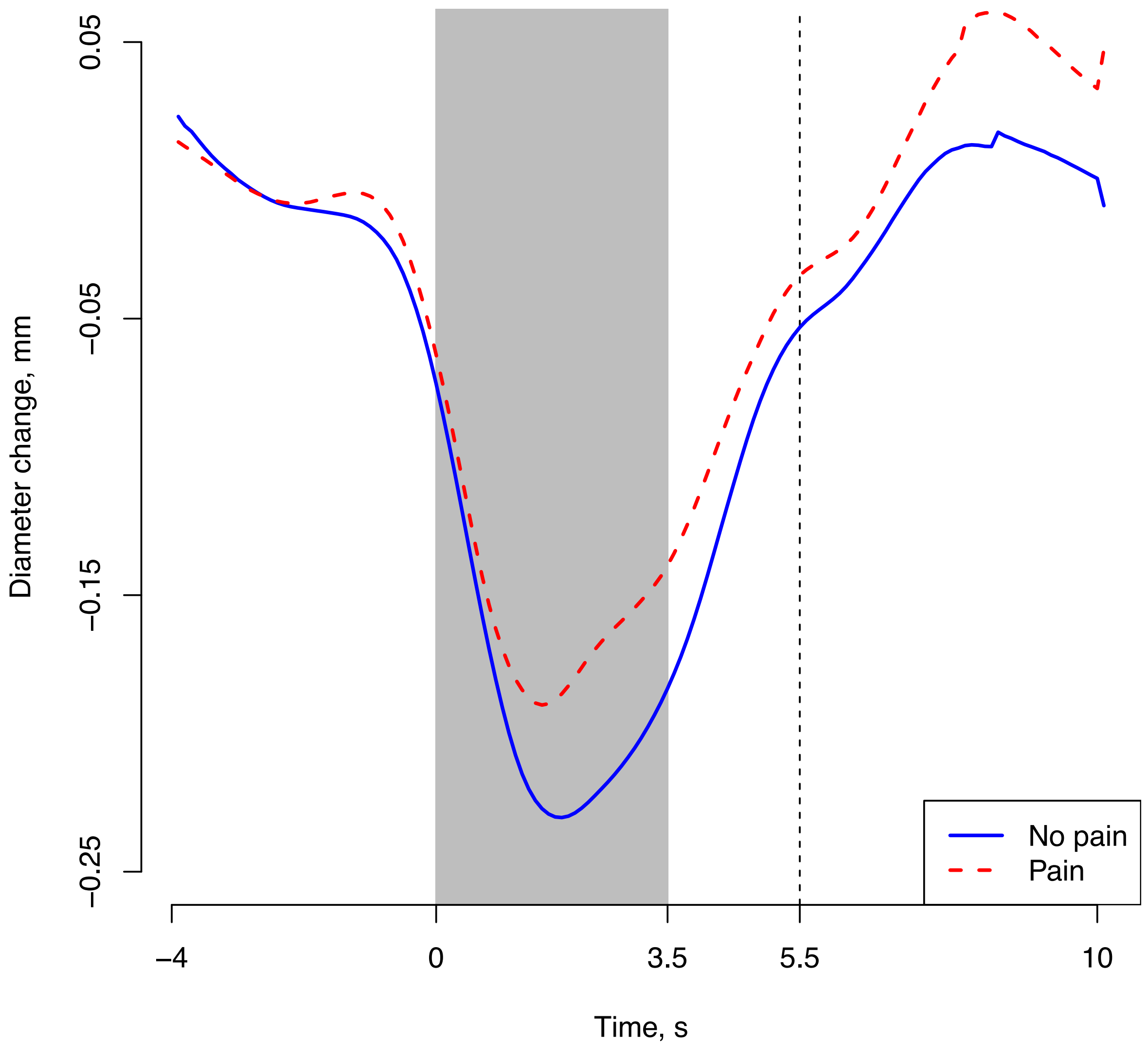

Supplementary figure 2a. Effect of stimulus type on pupil diameter

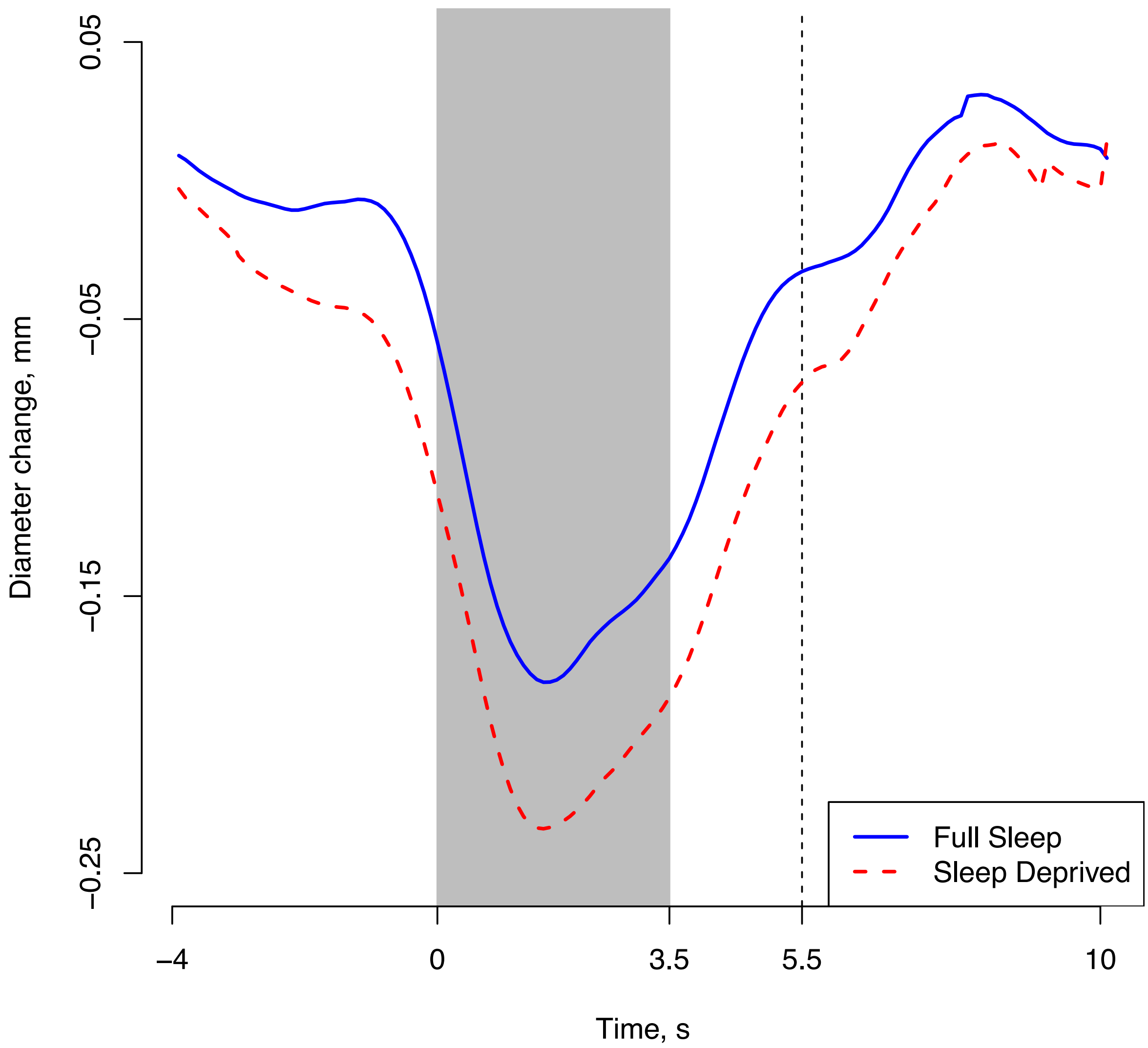

Supplementary figure 2b. Effect of sleep condition on pupil diameter

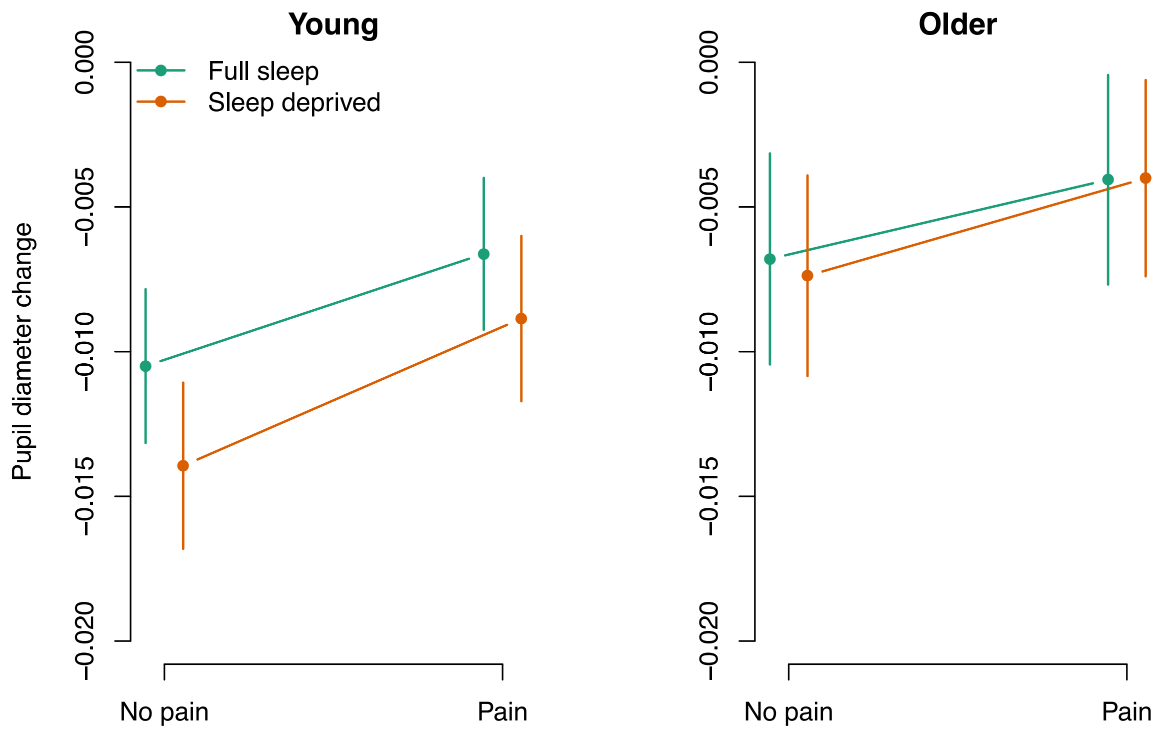

Supplementary figure 2c. Effect estimates and 95 % CI for pupil diameter change per condition and age group.

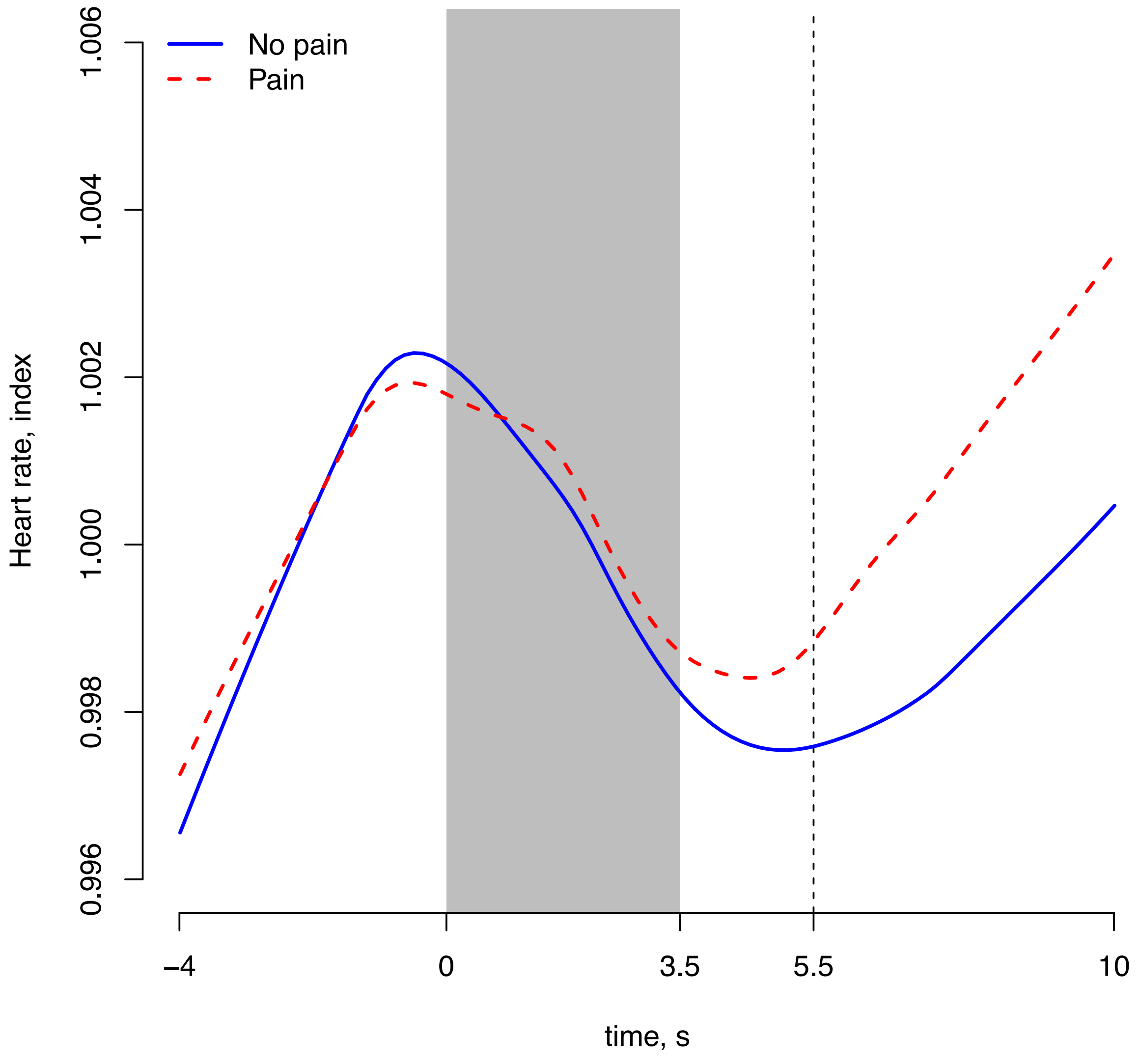

Supplementary figure 3a. Effects of stimulus on heart rate

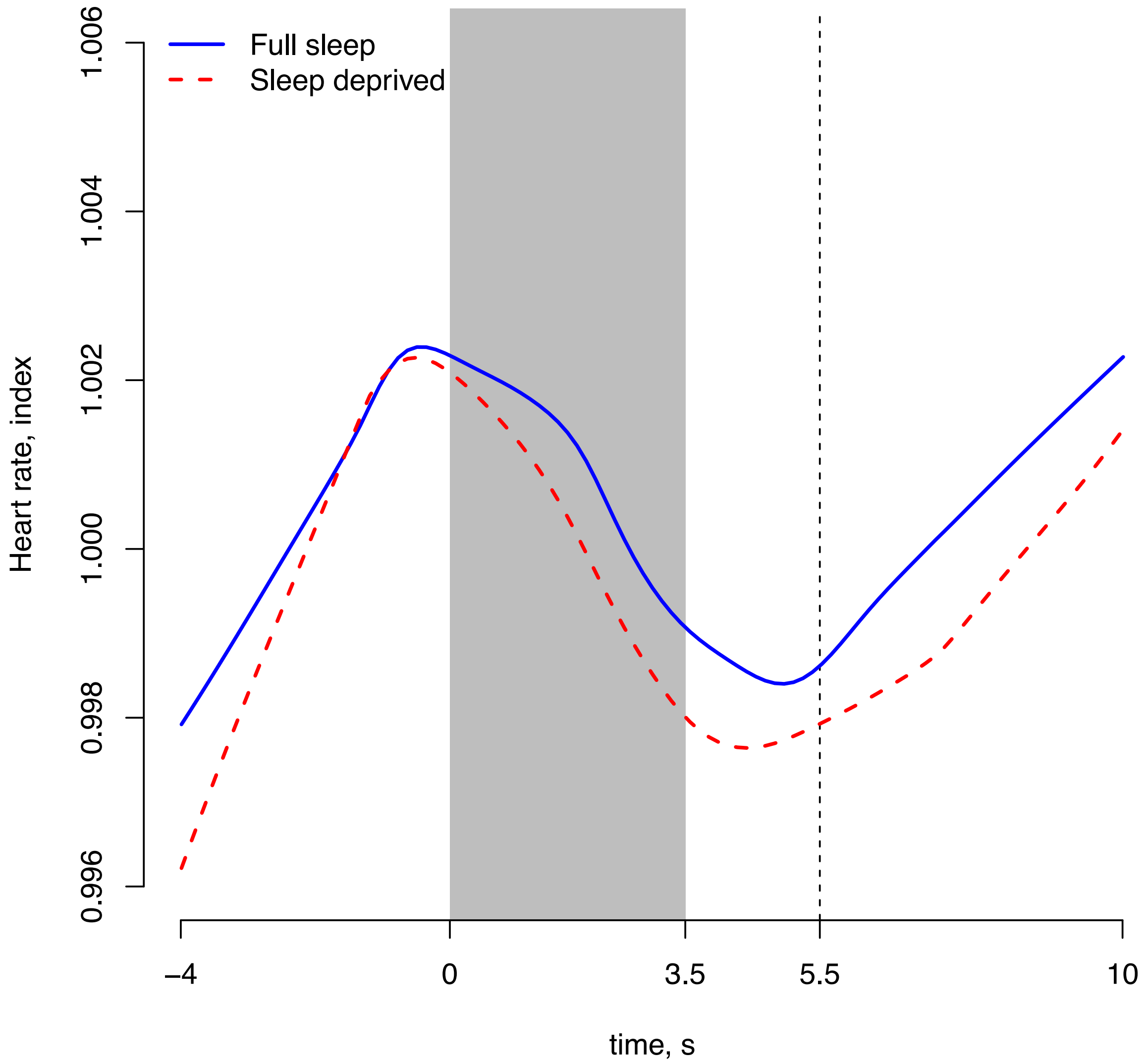

Supplementary figure 3b. Effects of sleep condition on heart rate

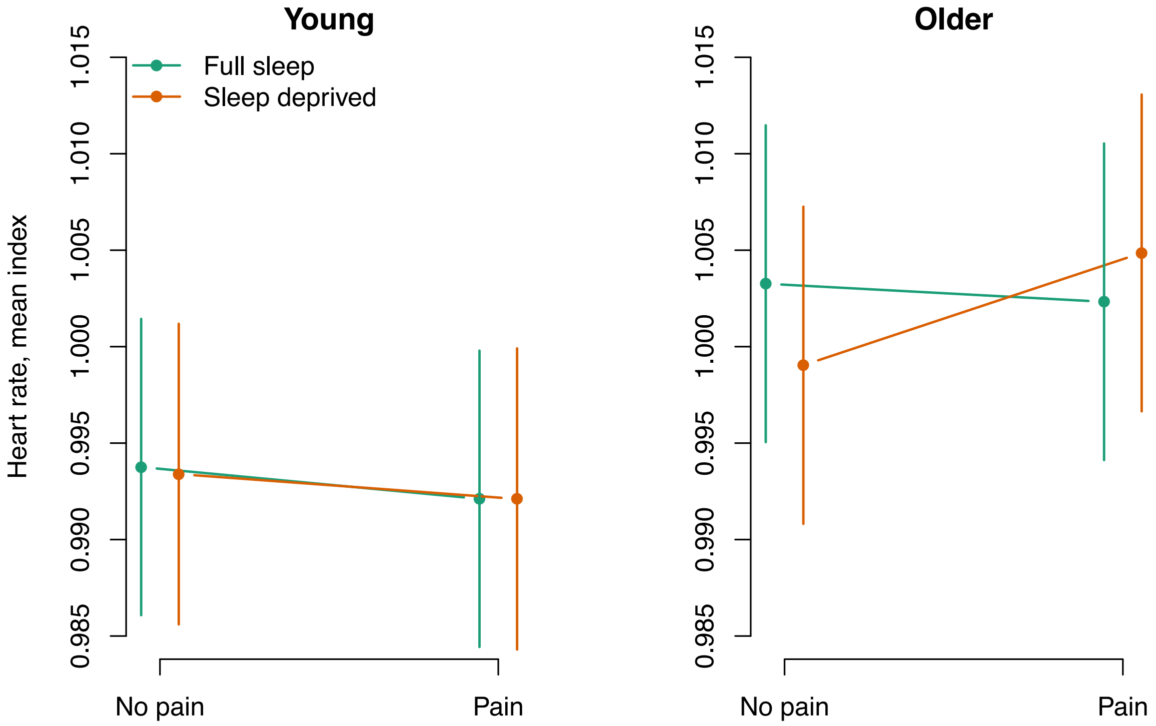

Figure 3c. Effect estimates and 95 % CI for heart rate per condition and age group.

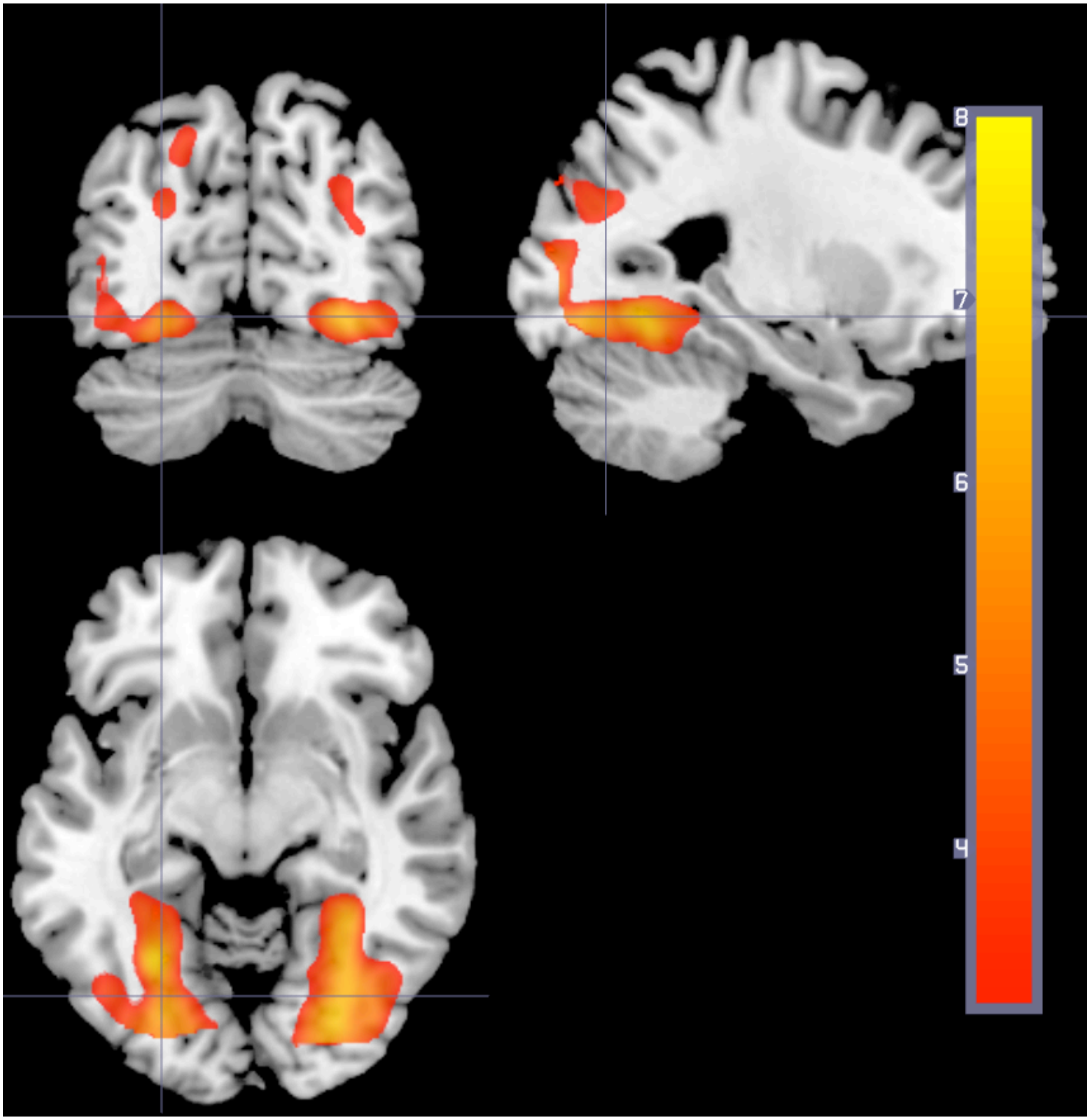

Supplementary figure 4. Older > young for the contrast no pain > baseline, 0.001 uncorrected for illustration. Color bar indicate T-values

set	set	cluster	cluster	cluster	cluster	peak	peak	peak	peak	peak			
p	c	p(FWE-corr)	p(FDR-corr)	equivk	p(unc)	p(FWE-corr)	p(FDR-corr)	T	equivZ	p(unc)	x	y	z {mm}
0	17	0	0	3613	0	0	0	7.28	6.68	0	-28	-60	-10
						0	0	6.21	5.82	0	-27	-78	-10
						0.004	0.004	5.4	5.14	0	-28	-86	2
		0	0	4770	0	0	0	6.81	6.31	0	27	-78	-9
						0	0	6.47	6.04	0	32	-50	-6
						0	0	6.45	6.02	0	32	-68	-10
		0.001	0.001	1218	0	0.033	0.024	4.87	4.67	0	-20	-86	38
						0.038	0.026	4.82	4.63	0	-21	-80	32
						0.499	0.23	3.97	3.86	0	-21	-66	40
		0.002	0.001	1145	0	0.084	0.054	4.6	4.43	0	33	-50	62
						0.121	0.073	4.49	4.33	0	33	-45	51
						0.717	0.348	3.77	3.67	0	26	-62	60
		0.045	0.018	536	0.007	0.162	0.088	4.4	4.25	0	51	10	28
		0.012	0.006	770	0.002	0.202	0.1	4.32	4.18	0	-42	4	27
						0.401	0.201	4.07	3.95	0	-51	9	20
						0.744	0.348	3.74	3.65	0	-54	6	40
		0.017	0.008	707	0.003	0.211	0.1	4.31	4.17	0	-54	-27	30
						0.526	0.239	3.95	3.84	0	-62	-18	30
		0.172	0.063	324	0.03	0.742	0.348	3.75	3.65	0	32	-3	51
						0.885	0.434	3.58	3.49	0	48	6	52
						0.886	0.434	3.58	3.49	0	42	-3	57
		0.901	0.515	50	0.363	0.751	0.348	3.74	3.64	0	40	36	15
		0.901	0.515	50	0.363	0.868	0.434	3.6	3.52	0	-18	-66	63
		0.837	0.485	69	0.285	0.888	0.434	3.57	3.49	0	-36	36	10
						0.939	0.52	3.48	3.4	0	-36	28	18
		0.957	0.647	29	0.495	0.919	0.479	3.52	3.44	0	64	-18	38
		0.827	0.485	72	0.275	0.96	0.587	3.43	3.35	0	-36	-40	42
		0.993	0.928	6	0.782	0.977	0.643	3.37	3.29	0	-34	-87	26
		0.995	0.928	4	0.829	0.979	0.644	3.36	3.29	0.001	-33	-57	63
		0.997	0.928	2	0.888	0.996	0.903	3.2	3.14	0.001	24	-57	21
		0.997	0.928	1	0.928	0.998	1	3.15	3.09	0.001	-10	-68	63

Supplementary table 1. Older > young for the contrast no pain > baseline

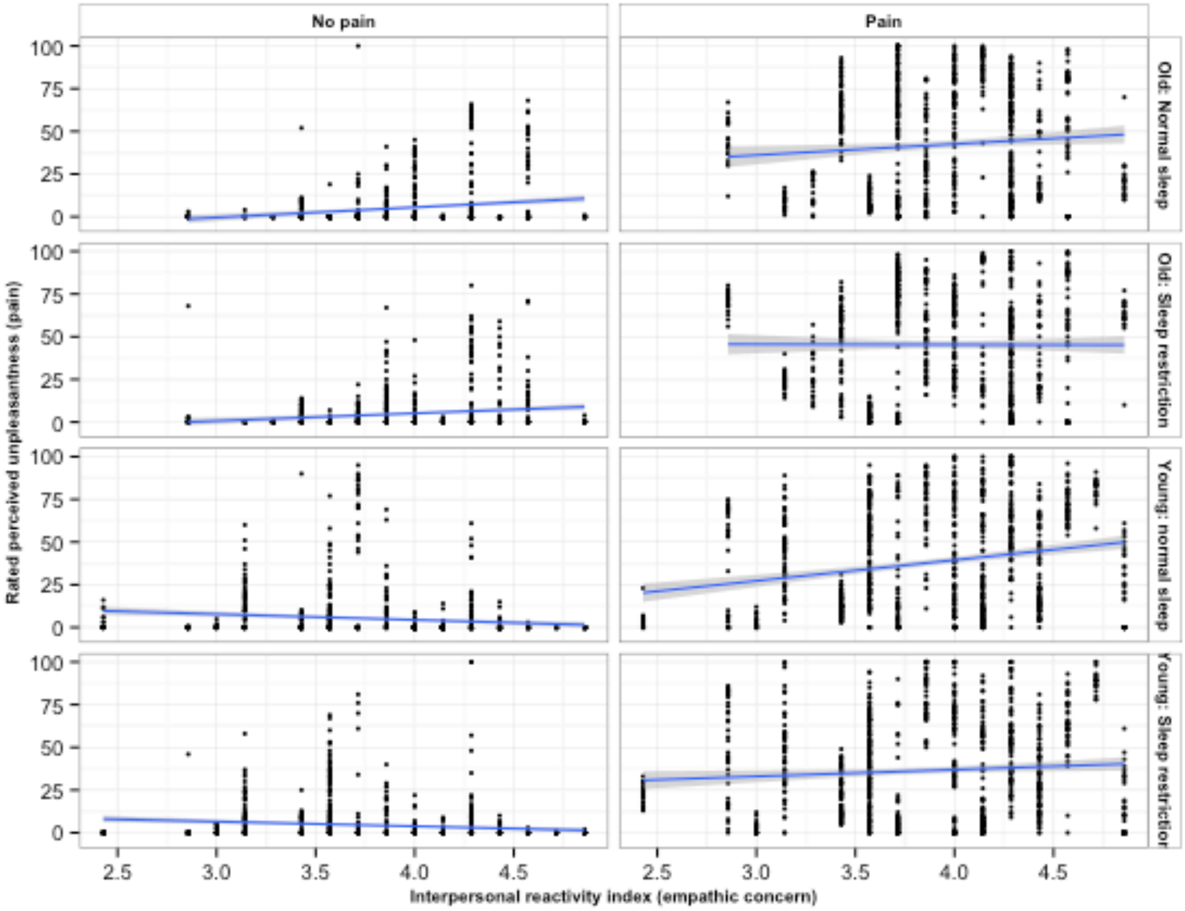

Supplementary figure 5. Relation between IRI-EC and rated unpleasantness, per stimulus type, age group, and sleep condition

Detailed fMRI tables

In the main manuscript, we provide peak level corrected statistics for the fMRI results. For completeness, we here provide corresponding tables (matching table 2-5 in main manuscript) including also cluster statistics.

Anatomical area (peak coordinate)		cluster	cluster	cluster	peak	peak	peak	peak
MRIcron (automated anatomical labeling)	MNI coords mm (x, y, z)	p(FWE-corr)	equivk	p(unc)	p(FWE-corr)	T	equivZ	p(unc)
Supramarginal (L)	-58, -27, 34	<0.001	12036	<0.001	<0.001	11.93	Inf	<0.001
Superior parietal (L)	-16, -68, 54				<0.001	9.44	7.78	<0.001
Inferior parietal (L)	-33, -46, 50				<0.001	9.02	7.53	<0.001
Inferior occipital (R)	33, -86, -4	<0.001	6294	<0.001	<0.001	10.15	Inf	<0.001
Middle occipital (R)	38, -82, 10				<0.001	8.78	7.39	<0.001
Middle occipital (R)	38, -74, 28				<0.001	8.32	7.1	<0.001
Precentral (L)	-51, 6, 30	<0.001	1599	<0.001	<0.001	9.31	7.71	<0.001
Rolandic operculum (L)	-52, 6, 9				0.006	5.43	5.02	<0.001
Superior medial frontal (L)	-4, 21, 44	<0.001	3203	<0.001	<0.001	8.75	7.37	<0.001
Middle cingulate cortex (L)	-4, 27, 38				<0.001	8.39	7.14	<0.001
Anterior cingulate cortex (L)	-2, 18, 27				0.029	5	4.67	<0.001
Middle frontal (L)	-26, 0, 52	<0.001	1141	<0.001	<0.001	8	6.89	<0.001
Insula (R)	32, 24, 4	<0.001	2101	<0.001	<0.001	7.69	6.68	<0.001
Inferior frontal operculum (R)	51, 9, 27				<0.001	7.41	6.49	<0.001
Inferior frontal operculum (R)	50, 15, 4				0.003	5.65	5.19	<0.001
Insula (L)	-34, 20, 6	<0.001	1054	<0.001	<0.001	7.52	6.56	<0.001
Inferior frontal, triangular part (L)	-44, 39, 6	<0.001	1034	<0.001	<0.001	6.6	5.92	<0.001
Inferior frontal, triangular part (L)	-42, 33, 27				<0.001	6.16	5.59	<0.001
Middle frontal (L)	-30, 48, 14				0.001	5.84	5.34	<0.001
Precentral (R)	30, -2, 52	<0.001	526	<0.001	<0.001	6.36	5.73	<0.001
Insula (L)	-36, -2, 15	0.007	43	0.144	0.002	5.79	5.3	<0.001
NA	-8, -24, -12	0.001	114	0.024	0.003	5.61	5.16	<0.001
Thalamus (L)	-12, -15, 9	0.007	45	0.136	0.006	5.43	5.02	<0.001

Supplementary table 2. Pain > no pain for all subjects across sleep conditions, thresholded at 0.05 FWE corrected and only clusters with > 20 voxels are reported. Anatomic labels are shown for peak coordinates and were defined using the automatic anatomic labelling in MRIcron.

Anatomical area (peak coordinate)		cluste	cluste	cluster	cluster	peak	peak	peak	peak	peak
MRIcron (automated anatomical labeling)	MNI coords mm (x, y, z)	p(FW	p(FDF	equivk	p(unc)	p(FWE-corr)	p(FDR-corr)	T	equivZ	p(unc)
Inferior parietal (L)	-30, -46, 45	<0.0(	<0.00	51828	<0.001	<0.001	<0.001	13.92	Inf	<0.001
Middle occipital (R)	33, -80, 32					<0.001	<0.001	13.59	Inf	<0.001
Superior parietal (L)	-20, -66, 51					<0.001	<0.001	13.05	Inf	<0.001
Inferior frontal operculum (R)	45, 9, 28	<0.0(	<0.00	9990	<0.001	<0.001	<0.001	12.16	Inf	<0.001
Insula (R)	34, 24, 4					<0.001	<0.001	10.57	Inf	<0.001
Precentral (R)	38, 2, 52					<0.001	<0.001	10.19	Inf	<0.001
Supplemental motor area (L)	-6, 9, 50	<0.0(	<0.00	4590	<0.001	<0.001	<0.001	12.09	Inf	<0.001
Supplemental motor area (R)	8, 14, 51					<0.001	<0.001	11.29	Inf	<0.001
Anterior cingulate cortex (R)	8, 15, 28					0.001	0.034	5.85	5.35	<0.001
NA	-8, -20, -8	<0.0(	<0.00	3950	<0.001	<0.001	<0.001	10.09	Inf	<0.001
Thalamus (L)	-14, -21, 6					<0.001	<0.001	8.8	7.4	<0.001
Thalamus (L)	-16, -28, 2					<0.001	<0.001	8.5	7.21	<0.001
Middle frontal (L)	-42, 34, 32	<0.0(	<0.00	3801	<0.001	<0.001	<0.001	7.95	6.85	<0.001
Middle frontal (L)	-32, 51, 14					<0.001	<0.001	7.47	6.53	<0.001
Middle frontal (L)	-40, 40, 22					<0.001	<0.001	7.38	6.47	<0.001
NA	14, 0, 2	0	0.38	25	0.226	0.004	0.079	5.62	5.17	<0.001
NA	-34, -3, -12	0	0.4	20	0.277	0.011	0.228	5.31	4.92	<0.001

Supplementary table 3. Pain > baseline for all subjects across sleep conditions, thresholded at 0.05 FWE corrected and only clusters with > 20 voxels are reported. Anatomic labels are shown for peak coordinates and were defined using the automatic anatomic labelling in MRIcron.

A										
Anatomical area (peak coordinate)		cluster	cluster	cluster	cluster	peak	peak	peak	peak	peak
MRIcron (automated anatomical labeling)	MNI coords mm (x, y, z)	p(FWE-corr)	p(FDR-corr)	equivk	p(unc)	p(FWE-corr)	p(FDR-corr)	T	equivZ	p(unc)
Angular (L)	-48, -74, 30	<0.001	<0.001	2857	<0.001	<0.001	0.001	6.4	5.98	<0.001
Middle occipital (L)	-40, -78, 36					0.001	0.003	5.8	5.48	<0.001
Angular (L)	-40, -62, 32					0.143	0.14	4.33	4.19	<0.001
Calcarine (L)	-2, -87, 14	<0.001	<0.001	9176	<0.001	0.005	0.017	5.27	5.02	<0.001
Cuneus (L)	-10, -93, 14					0.007	0.019	5.18	4.94	<0.001
Calcarine (L)	-2, -63, 16					0.017	0.039	4.95	4.74	<0.001
Angular (R)	48, -69, 32	0.073	0.083	589	0.017	0.025	0.049	4.84	4.65	<0.001
Superior frontal (R)	30, 27, 52	0.023	0.032	860	0.005	0.028	0.049	4.81	4.62	<0.001
Middle frontal (R)	26, 42, 42					0.327	0.257	4.04	3.92	<0.001
Middle temporal (L)	-56, -48, -6	0.012	0.022	1025	0.003	0.078	0.092	4.52	4.36	<0.001
Middle temporal (L)	-51, -42, 0					0.672	0.488	3.7	3.6	<0.001
Middle temporal (L)	-56, -36, 10					0.891	0.629	3.44	3.37	<0.001
Middle frontal (L)	-27, 38, 45	0.363	0.309	250	0.099	0.256	0.202	4.13	4.01	<0.001
Superior frontal (L)	-21, 44, 44					0.745	0.488	3.62	3.53	<0.001
Superior frontal (L)	-22, 36, 36					0.958	0.847	3.3	3.23	0.001
Superior temporal (R)	64, -9, 8	0.269	0.287	311	0.069	0.457	0.362	3.9	3.79	<0.001
Rolandic operculum (R)	51, -8, 20					0.957	0.847	3.3	3.23	0.001
Postcentral (R)	64, -6, 27	0.356	0.309	254	0.097	0.644	0.488	3.72	3.63	<0.001
Precentral (R)	20, -22, 66	0.862	0.777	54	0.435	0.653	0.488	3.71	3.62	<0.001
Precentral (R)	30, -22, 64					0.939	0.776	3.35	3.28	0.001
Medial frontal orbital (R)	2, 54, -8	0.694	0.542	111	0.26	0.676	0.488	3.69	3.6	<0.001
Cerebelum (R)	12, -54, -8	0.628	0.494	134	0.217	0.707	0.488	3.66	3.57	<0.001
Precentral (R)	45, -10, 52	0.478	0.357	193	0.143	0.715	0.488	3.65	3.56	<0.001
Parahippocampal (L)	-20, -38, -8	0.476	0.357	194	0.142	0.717	0.488	3.65	3.56	<0.001
Parahippocampal (L)	-27, -36, -10					0.791	0.508	3.57	3.49	<0.001
Superior temporal (L)	-54, -15, 2	0.935	0.918	26	0.6	0.732	0.488	3.64	3.55	<0.001
Superior temporal (R)	46, -32, 15	0.795	0.67	77	0.348	0.784	0.508	3.58	3.5	<0.001
Superior temporal (R)	57, -2, -8	0.942	0.918	23	0.624	0.869	0.602	3.48	3.4	<0.001
Precentral (R)	39, -15, 38	0.93	0.918	28	0.584	0.966	0.869	3.28	3.21	0.001
Precentral (R)	36, -15, 48					0.989	0.989	3.16	3.1	0.001
B										
Anatomical area (peak coordinate)	MNI coords mm (x, y, z)	cluster	cluster	cluster	cluster	peak	peak	peak	peak	peak
MRIcron (automated anatomical labeling)		p(FWE-corr)	p(FDR-corr)	equivk	p(unc)	p(FWE-corr)	p(FDR-corr)	T	equivZ	p(unc)
Anterior cingulate cortex (R)	9, 24, 26	0.634	0.491	122	0.152	0.334	0.308	4.15	4.03	<0.001
Postcentral (L)	-39, 22, 50	0.727	0.491	98	0.196	0.604	0.351	3.89	3.78	<0.001
Postcentral (L)	-51, -26, 57	0.968	0.853	25	0.52	0.984	0.929	3.34	3.27	0.001
C										
Anatomical area (peak coordinate)	MNI coords mm (x, y, z)	cluster	cluster	cluster	cluster	peak	peak	peak	peak	peak
MRIcron (automated anatomical labeling)		p(FWE-corr)	p(FDR-corr)	equivk	p(unc)	p(FWE-corr)	p(FDR-corr)	T	equivZ	p(unc)
Fusiform (L)	-24, -75, -10	<0.001	<0.001	15740	<0.001	<0.001	<0.001	7.93	7.19	<0.001
Fusiform (L)	-28, -60, -10					<0.001	<0.001	7.56	6.9	<0.001
Fusiform (R)	28, -66, -9					<0.001	<0.001	7.28	6.69	<0.001
Precentral (R)	58, 2, 40	0.178	0.123	311	0.03	0.081	0.064	4.62	4.45	<0.001
Precentral (R)	63, 9, 26					0.933	0.623	3.51	3.43	<0.001
Superior occipital (R)	24, -81, 40	0.139	0.107	348	0.023	0.083	0.064	4.61	4.44	<0.001
Superior occipital (R)	32, -76, 45					0.871	0.537	3.61	3.53	<0.001
Superior parietal (R)	34, -45, 62	<0.001	0.001	1463	<0.001	0.116	0.076	4.51	4.35	<0.001
Inferior parietal (R)	28, -48, 54					0.183	0.102	4.37	4.22	<0.001
Postcentral (R)	32, -33, 51					0.802	0.485	3.7	3.6	<0.001
Middle frontal (R)	44, -3, 54	0.008	0.011	817	0.001	0.12	0.076	4.5	4.34	<0.001
Precentral (R)	34, -2, 50					0.334	0.154	4.15	4.03	<0.001
Superior frontal (R)	32, -4. 62					0.693	0.375	3.81	3.71	<0.001
Postcentral (L)	-62, -15, 30	0.009	0.011	799	0.001	0.282	0.145	4.22	4.08	<0.001
Supramarginal (L)	-58, -26, 26					0.298	0.145	4.2	4.07	<0.001
Postcentral (L)	-51, -22, 33					0.903	0.587	3.57	3.48	<0.001
Calcarine (R)	21, -54, 10	0.128	0.107	360	0.021	0.304	0.145	4.19	4.06	<0.001
Lingual (R)	6, -60, 9					0.955	0.649	3.46	3.38	<0.001
NA	9, -27, -4	0.062	0.064	472	0.01	0.479	0.228	4	3.89	<0.001
NA	-3, -45, 8					0.857	0.537	3.63	3.54	<0.001
NA	-6, -28, -8					0.906	0.587	3.56	3.48	<0.001
Superior parietal (L)	-33, -57, 63	0.935	0.857	39	0.415	0.707	0.38	3.79	3.69	<0.001
Inferior parietal (L)	-34, -40, 48	0.86	0.755	63	0.297	0.806	0.485	3.69	3.6	<0.001
NA	24, -26, -4	0.953	0.857	32	0.462	0.852	0.537	3.64	3.55	<0.001
Inferior frontal operculum (L)	-44, 6, 30	0.275	0.178	248	0.049	0.866	0.537	3.62	3.53	<0.001
Precentral (L)	-50, 3, 34					0.868	0.537	3.62	3.53	<0.001
Postcentral (R)	66, -12, 22	0.955	0.857	31	0.47	0.884	0.554	3.59	3.51	<0.001
Inferior frontal operculum (L)	-52, 9, 20	0.684	0.575	109	0.174	0.914	0.598	3.55	3.46	<0.001
Precentral (L)	-44, -3, 58	0.816	0.705	75	0.256	0.932	0.623	3.51	3.43	<0.001
Precentral (L)	-40, -4, 51					0.975	0.702	3.39	3.32	<0.001
Superior temporal (L)	-46, -8, -6	0.943	0.857	36	0.434	0.95	0.649	3.47	3.39	<0.001
Middle cingulate cortex (R)	12, -20, 44	0.968	0.857	25	0.52	0.961	0.649	3.44	3.36	<0.001
Postcentral (R)	60, -12, 40	0.774	0.675	86	0.225	0.967	0.672	3.42	3.35	<0.001
Posterior cingulate cortex (L)	-8, -36, 34	0.966	0.857	26	0.511	0.971	0.686	3.41	3.33	<0.001
Middle frontal (R)	33, 38, 40	0.957	0.857	30	0.477	0.979	0.724	3.37	3.3	<0.001

Supplementary table 4. Effects of age. All tables show data thresholded at 0.001 uncorrected, only clusters with > 20 voxels are shown. Anatomic labels are shown for peak coordinates and were defined using the automatic anatomic labelling in MRIcron. ***A.*** *The contrast older > young for pain > no pain,* ***B.*** *The contrast young > older for pain > baseline.* ***C.*** *The contrast older > young for pain > baseline.*

Anatomical area (peak coordinate)	MNI coors mm (x, y, z)	cluster	cluster	cluster	cluster	peak	peak	peak	peak	peak
MRIcron (automated anatomical labeling)		p(FWE-corr)	p(FDR-corr)	equivk	p(unc)	p(FWE-corr)	p(FDR-corr)	F	equivZ	p(unc)
Insula (L)	-45, -8, 3	0.201	0.198	260	0.028	0.624	0.574	16.79	3.8	<0.001
Insula (R)	46, -4, 2	0.455	0.268	159	0.077	0.929	0.849	14.2	3.49	<0.001
Superior temporal (R)	46, -3, -8					0.95	0.849	13.88	3.45	<0.001

*Supplementary table 5.Table showing the interaction contrast age group*sleep condition for pain > baseline, thresholded at 0.001 uncorrected, only clusters with > 20 voxels are shown. Anatomic labels are shown for peak coordinates and were defined using the automatic anatomic labelling in MRIcron.*
